# Synthesis and Solution Properties of a Novel Hyperbranched Polymer Based on Chitosan for Enhanced Oil Recovery

**DOI:** 10.3390/polym12092130

**Published:** 2020-09-18

**Authors:** Qingyuan Chen, Zhongbin Ye, Lei Tang, Tao Wu, Qian Jiang, Nanjun Lai

**Affiliations:** 1 School of Chemistry and Chemical Engineering of Southwest Petroleum University, Chengdu 610500, Sichuan, China; 201911000046@stu.swpu.edu.cn (Q.C.); 201811000045@stu.swpu.edu.cn (L.T.); 201921000222@stu.swpu.edu.cn (Q.J.); 2 Oil & Gas Field Applied Chemistry Key Laboratory of Sichuan Province, Chengdu 610500, Sichuan, China; 3 Sanjiang Aerospace Jianghe Chemical Technology Co., Ltd., Yuan’an 444200, Hubei, China; 201821000247@stu.swpu.edu.cn; 4 State Key Laboratory of Oil and Gas Reservoir Geology and Exploitation, Chengdu University of Technology, Chengdu 610059, Sichuan, China

**Keywords:** polymer flooding, synthesis, hyperbranched structure, chitosan, solution properties, enhanced oil recovery

## Abstract

A new type of chitosan-modified hyperbranched polymer (named HPDACS) was synthesized through the free-radical polymerization of surface-modified chitosan with acrylic acid (AA) and acrylamide (AM) to achieve an enhanced oil recovery. The optimal polymerization conditions of HPDACS were explored and its structure was characterized by Fourier-transform infrared spectroscopy, hydrogen nuclear magnetic resonance, and environmental scanning electron microscopy. The solution properties of HPDACS in ultrapure water and simulated brine were deeply studied and then compared with those of partially hydrolyzed polyacrylamide (HPAM) and a dendritic polymer named HPDA. The experimental results showed that HPDACS has a good thickening ability, temperature resistance, and salt resistance. Its viscosity retention rate exceeded 79.49% after 90 days of aging, thus meeting the performance requirements of polymer flooding. After mechanical shearing, the viscosity retention rates of HPDACS in ultrapure water and simulated brine were higher than those of HPAM and HPDA, indicating its excellent shear resistance and good viscoelasticity. Following a 95% water cut after preliminary water flooding, 0.3 pore volume (PV) and 1500 mg/L HPDACS solution flooding and extended water flooding could further increase the oil recovery by 19.20%, which was higher than that by HPAM at 10.65% and HPDA at 13.72%. This finding indicates that HPDACS has great potential for oil displacement.

## 1. Introduction

As a tertiary oil recovery technology, polymer flooding has become one of the most mature and effective strategies for enhanced oil recovery (EOR) [1,2,3,4,5]. This process improves the water–oil mobility ratio by increasing the viscosity of injected water, thereby increasing the flow resistance of the displacement phase in high-permeability reservoirs, forcing the displacement phase to move into a relatively low-permeability layer, and increasing the water-flooding coverage and reservoir recovery.

The commonly used polymers in oil fields are polyacrylamide (PAM) [6,7], partially hydrolyzed polyacrylamide (HPAM) [8,9,10], and their derivatives. When pumped through blast holes and porous media, these materials are strongly sheared and stretched, causing the irreversible degradation of polymer molecular chains, and reducing their molecular size and corresponding weight-average molecular weight and viscosity [11,12,13,14,15]. Poor temperature and salt resistance prevent the application of polymers in oil production [16].

The hyperbranched polymer has a highly branched 3D structure, a number of terminal functional groups, high chemical reactivity, and good solubility and thus, has received increasing interest [17,18,19,20,21,22]. The structural properties of hyperbranched macromolecules can substantially improve the shear resistance of polymers [23,24]. However, hyperbranched polymers are difficult to separate from the solution [25]. Hyperbranched polymers have abundant branched chains that can form a dense network structure and allow for a high viscosity retention rate after shearing [26,27,28,29]. Therefore, a sufficient resistance factor and residual resistance factor can be established in porous media and this strategy shows application potential in oil displacement.

As a natural and renewable raw material with abundant sources and no pollution, polysaccharides have good temperature and shear resistance [30]. Many studies have been published on chitosan, a white powdery solid that is nontoxic, harmless, and biodegradable and has a molecular structure containing highly reactive –OH and –NH_2._ Its chemical modification mainly includes acylation [31], alkylation [32], esterification [33], etherification [34,35], and cross-linking [36,37]. Given its excellent biocompatibility and biodegradability, chitosan and its derivatives are widely used in medical materials, food, wastewater treatment, and other fields. However, its application as a chemical oil displacement agent in EOR has been rarely reported. The highly active group on the chitosan surface provides convenience for its application. Therefore, introducing chitosan into a high-molecular polymer chain as an oil displacing agent may improve the biodegradability of the polymer to reduce environmental pollution.

In this study, a novel hyperbranched polymer based on modified chitosan (HPDACS) was successfully synthesized using acrylic acid (AA), acrylamide (AM), and chitosan modified by a branched monomer (DACS). HPDACS was characterized through hydrogen nuclear magnetic resonance (^1^ HNMR), Fourier-transform infrared (FT-IR) spectroscopy, environmental scanning electron microscopy (ESEM), and static light scattering/dynamic light scattering (SLS/DLS). Solution properties, including solubility, thickening ability, temperature and salt resistance, shear resistance, antiaging performance, and viscoelasticity, were compared among HPDACS, the dendritic polymer (HPDA), and HPAM. Their EOR performances were evaluated by core-flooding experimentation.

## 2. Experimental Section

### 2.1. Experimental Materials

Ethylenediamine, methyl acrylate, petroleum ether, ethyl acetate, methanol, maleic anhydride, AA, AM, acetic acid, trichloromethane, sodium hydroxide (NaOH), N,N-dimethylformamide (DMF), ammonium persulfate ((NH_4_)_2_S_2_O_8_), sodium bisulfite (NaHSO_3_), silica gel, sodium chloride (NaCl), magnesium chloride (MgCl_2_), and calcium chloride (CaCl_2_) were purchased from Chengdu Kelong Chemical Reagent Co., Ltd., Chengdu, China. Chitosan was purchased from Shanghai Aladdin Chemical Reagent Co., Ltd., Shanghai City, China. HPAM and HPDA were laboratory-made. The crude oil used in the oil displacement experiment came from an oilfield in Xinjiang with a viscosity of 12.5 mPa·s at 60 °C.

### 2.2. Synthesis of HPDACS

#### 2.2.1. Synthesis of Branched Monomers

**Figure 1:**
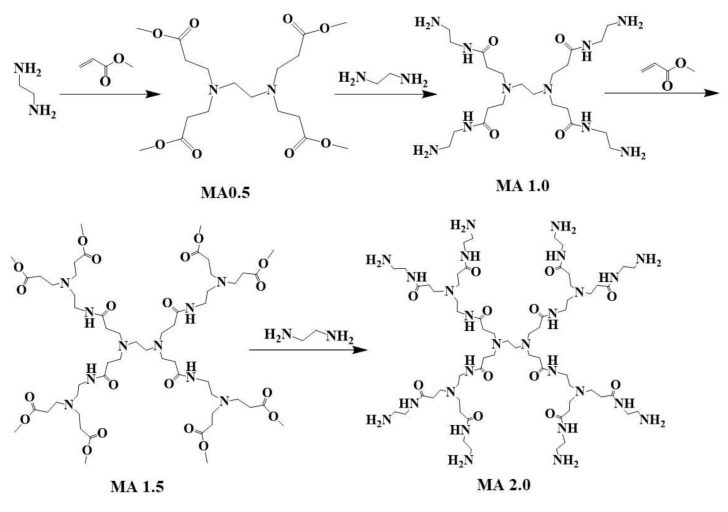
Reaction scheme of branched monomers MA0.5, MA1.0, MA1.5 and MA2.0.

(1) Under the protection of an ice bath and nitrogen and with methanol as the solvent, methyl acrylate solution was dripped into ethylenediamine solution using a constant-pressure-dropping funnel at a rate of one to two drops per second. After the reaction was completed, the solution stayed in the ice bath for 30 min, and the reaction system was placed at 25 °C to detect the reaction process at all times through a spot plate. After the reaction was completed, the solvent methanol and excess methyl acrylate were removed through vacuum distillation at 45 °C. The product was purified by column chromatography with silica gel as the adsorbent and ethyl acetate/petroleum ether of 1:3 volume ratio as the eluent, detected by spot plate at all times to determine the product. Finally, the eluent was removed through vacuum distillation to obtain a light yellow liquid (named MA0.5) with a yield of 95.2%. (2) Under the same conditions, ethylenediamine solution was uniformly dropped into MA0.5 solution to obtain a light yellow viscous liquid MA1.0 with a yield of 93.6%. (3) Similar to step (1), methyl acrylate solution was dropped into MA1.0 solution at a uniform speed to obtain the yellow viscous liquid MA1.5 with a yield of 81.2%. (4) Similar to step (2), the ethylenediamine solution was dropped into MA1.5 solution at a constant speed to finally obtain a dark yellow viscous liquid MA2.0 with a yield of 91.7%. The reaction scheme of MA2.0 is shown in Figure 1 (details of synthesis and FT-IR and ^1^ HNMR characterization of branched monomers are shown in the Supplementary Materials).

#### 2.2.2. Synthesis of NCS

Surface-modified chitosan (named NCS) was prepared as follows. (1) In brief, 1.0 g of reprecipitated and purified chitosan were added in 100 mL of diluted acetic acid solution, dissolved completely in 1–2 h under the action of a magnetic stirrer, and diluted with 50 mL of methanol solution. (2) Under the protection of nitrogen, methanol solution of methyl acrylate was dropwise added to the chitosan solution with a constant-pressure-funnel at a rate of one drop per second while maintaining the reaction temperature at 25 °C for 3 days. (3) After the reaction, the product was distilled to remove unreacted methyl acrylate and solvent methanol, purified by dialysis, and freeze-dried to obtain pure NCS.

#### 2.2.3. Synthesis of MACS

Surface-modified chitosan (named MACS) was prepared as follows. (1) In brief, 0.3 g of NCS was placed in a three-port flask and added with 50 mL of methanol solution under the action of a magnetic stirrer. (2) Under the protection of nitrogen, MA2.0 was dropwise added to NCS methanol solution by a constant-pressure-funnel at a rate of one drop per second with the reaction temperature maintained at 25 °C for 2 days. (3) After the reaction, the product was distilled to remove the solvent methanol, dispersed in a solution of 0.2 mol/L NaOH, purified by dialysis, and freeze-dried to obtain pure MACS.

#### 2.2.4. Synthesis of DACS

DACS was prepared as follows. (1) In brief, 0.5 g of MACS was added in 30 mL of DMF solution. (2) Under the protection of nitrogen, maleic anhydride solution was dropwise added to MACS solution by a constant-pressure-funnel at a rate of one drop per second with the reaction temperature maintained at 70 °C for 6 h to obtain a brown transparent solution. (3) After the reaction, the crude product was dropwise added to a certain amount of trichloromethane solution, mixed with a magnetic stirrer to precipitate light yellow powder particles, and dissolved in DMF. (4) After repeated precipitation with trichloromethane, the solution was rapidly filtered to obtain pure DACS.

#### 2.2.5. Synthesis of HPDACS

**Figure 2:**
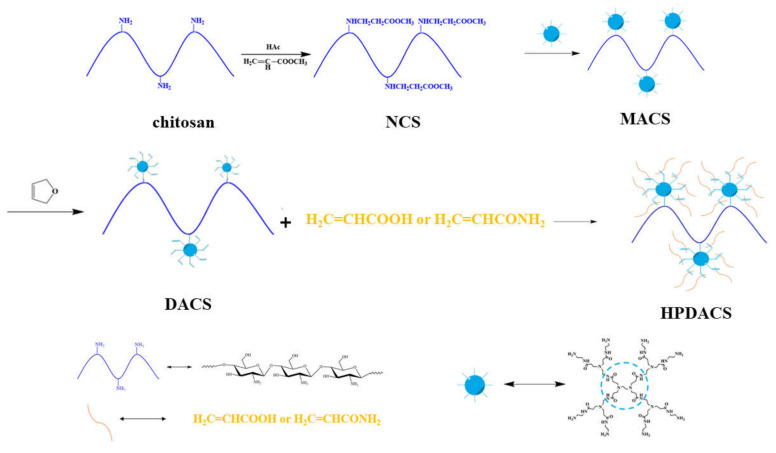
Synthesis schematic route of NCS (modified chitosan), MACS (further modified chitosan), DACS (branched monomer modified chitosan) and HPDACS (chitosan modified hyperbranched polymer).

HPDACS was prepared as follows. (1) A certain amount of AA was dissolved in ultrapure water, and the pH of the solution was adjusted and maintained at 7 using a NaOH solution. (2) A certain amount of AM and DACS were added in nitrogen under water bath stirring at constant temperature, and the initiator (n (NaHSO_3_: n(NH_4_)_2_S_2_O_8_ = 1: 1) was then added to react at a constant temperature for 6 h. (3) After reaction completion, the reaction products were sliced, dissolved, purified through dialysis, and freeze-dried to obtain a pure white powder named HPDACS. The synthesis route is shown in Figure 2.

### 2.3. Characterization

The infrared spectra of chitosan, NCS, MACS, DACS, and HPDACS were measured by a WQF-520 infrared spectrometer (Beijing Rayleigh Analytical Instrument Corporation, Beijing, China) by the KBr (spectral pure) tableting method. Chitosan, NCS, MACS, and DACS were tested by a DX-2700 X-ray diffractometer produced by Dandong Haoyuan Instruments Co., Dandong, China. The scanning range was controlled to be 2θ = 5~30°, and the stepping angle was 0.02°. The mass percentages of C and N elements in NCS, MACS, and DACS were determined using a Var10EL-III elemental analyzer, which was purchased from Elementar Analysensysteme GmbH, Hanau, Germany. Using deuterium-substituted trichloromethane as the solvent, HPDACS was characterized by a 400-MHz NMR system (Bruker Corporation, Billerica, MA, USA). A BI-200SM SLS/DLS instrument (purchased from Brookhaven Instruments, Austin, TX, USA) was used to measure the weight-average molecular weight (*M*_W_) and hydrodynamic radius (*R*_h_) of different polymers. The morphology of each sample in ultrapure water was observed using a Quanta 450 ESEM system purchased from FEI Company, Hillsboro, OR, USA.

### 2.4. Solution Properties

The solution properties of polymers, mainly including thickening ability, temperature and salt resistance, anti-shearing performance, and rheological viscoelasticity, have been widely studied. In this work, the solution performance of HPDACS in simulated brine and ultrapure water were systematically studied and compared with those of HPAM and HPDA (structure and synthesis methods are previously described [36,37]) to determine whether HPDACS meets the basic performance requirements of polymer flooding. Unless otherwise specified, the resistivity of the ultrapure water used in the experiment was 18.25 MΩ·cm. The ionic composition of the simulated brine is shown in Table 1, and its total dissolved solids (TDS) is 10,600 mg/L.

#### 2.4.1. Solubility

Solubility is the first thing to consider in polymer application. Ionic polymers transform as polymer electrolytes after dissolution in simulated brine or ultrapure water. With the continuous swelling and dissolution of the polymer, the viscosity and conductivity of the solution increase continuously until the solution is completely dissolved, and the viscosity and conductivity of the solution tend to stabilize. Therefore, the solubility of the polymer can be quantitatively judged according to the conductivity method. Here, the conductivity of a 1500 mg/L polymer solution at 25 °C was measured by a DDS-307 conductivity meter purchased from Shanghai Yoke Instrument Co., Shanghai, China.

#### 2.4.2. Thickening Ability

Thickening ability is one of the most important properties of a polymer acting as a displacement agent. The apparent viscosities of HPAM, HPDA, and HPDACS at different concentrations were measured by a DV-III rheometer (Brookfield Co., Middleboro, MA, USA) at 60 °C.

#### 2.4.3. Stability Experiments

The stabilities of HPAM, HPDA, and HPDACS were evaluated. The effects of temperature, salinity, mechanical shear strength, and aging time on the solution viscosity in ultrapure water and simulated brine were also investigated. Apparent viscosity was measured with a DV-III rheometer, and the influence of mechanical shear strength on the apparent viscosity of the solution was investigated with a WT-VSA2000B Waring mixer (Beijing Exploration Engineering Research Institute of China Geological Survey, Beijing, China) at shear strengths of 3500, 7000, and 11,500 rpm for 20 s.

#### 2.4.4. Rheology and Viscoelasticity

The rheological property of a polymer enables the random coils of its molecules to deform and migrate when passing through porous media without strong shear and tensile degradation, thus effectively avoiding serious viscosity loss for the polymer. Furthermore, the blind residual oil in the formation is “pulled and dragged” by the viscoelasticity of the polymer solution; a material with high viscoelasticity can carry a large amount of oil and produce a high recovery ratio [38,39,40]. In laboratory research, elastic modulus (*G*′) and viscous modulus (*G*″) are often used to describe the elasticity and viscosity of polymer solution.

The rheological property, elastic modulus, and viscous modulus of polymer solution (1500 mg/L) in ultrapure water and simulated brine were measured by a MARS III rotary rheometer (HAAKE Technik GmbH, Vreden, Germany). The temperature was controlled at 60 °C, the shear rate was 1–1000–1 s^‒1^, the scanning frequency was 0.01–40 Hz, and the scanning stress was 0.10 Pa. The geometric model of the test system is the cylinder model in steady-state shear test and the cone plate in viscoelasticity test.

### 2.5. Oil Displacement Capability of Polymers

After the polymer solution was injected into porous media, the water–oil mobility ratio in the flooding process was generally improved by increasing the water-phase viscosity and the water content in the produced fluid was reduced, thus improving the crude oil recovery ratio.

Polymer EOR is calculated according to Equation (1):

(1) $$\mathrm{EOR}=E_{2}-E_{1}$$

where EOR is the recovery factor of polymer flooding, vol.%; E_2_ is the total oil recovery combining the preliminary water flooding, polymer flooding and extended water flooding, vol.% and E_1_ is the oil recovery of the preliminary water flooding, vol.%

Different types of HPAM, HPDA, and HPDACS polymer solutions have varying abilities to establish flow resistance due to their different molecular structures. Hence, the feasibility of using HPDACS for EOR in reservoirs was analyzed by studying the displacement performance of HPAM, HPDA, and HPDACS.

The basic parameters of a one-dimensional sandpack model for the oil displacement experiment are shown in Table 2. The whole oil displacement process can be roughly divided into three stages: (1) preliminary water displacement until the water content of the produced solution reaches 95%, (2) injection of 0.3 pore volume (PV) polymer solution, and (3) extended water displacement until the water content of the produced solution reaches 95%.

**Figure 3:**
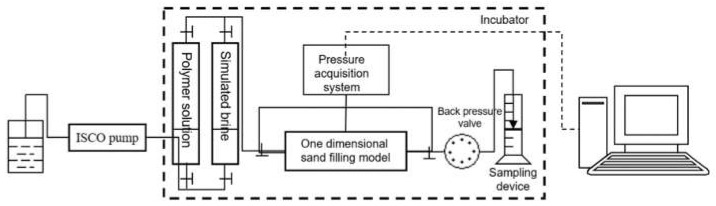
Flow chart of the polymer displacement experiment.

The experimental flow chart is shown in Figure 3. The sandpack model consisted of an HXH-100B double-cylinder, constant-flow, constant-pressure pump; 1000 mL piston containers and pressure sensors with measuring ranges of 0–1 and 0–5 MPa. The pipeline has an inner diameter of 3 mm, and the one-dimensional sandpack model at a size of 25 × 2.5 cm has gas/liquid collectors. Both were purchased from Hai’an Petroleum instrument Co., Chengdu, China.

## 3. Results and Discussion

### 3.1. Optimal Synthesis Conditions of HPDACS

The biochemical oxygen demand (BOD) of a polymer refers to the amount of oxygen consumed by the microorganisms in the solution when they decompose the polymer. This value can objectively reflect the degradability of the polymer. In general, a high BOD indicates a strong degradation ability for a polymer. The 5-day BOD, BOD_5_, is often used to describe whether the polymer has been completely decomposed by microorganisms. In this paper, an AER-208 microbial degradation respirator (Challenge Industrial Co., Middleboro, MA, USA) was used to determine the BOD_5_ of HPDACS.

**Figure 4:**
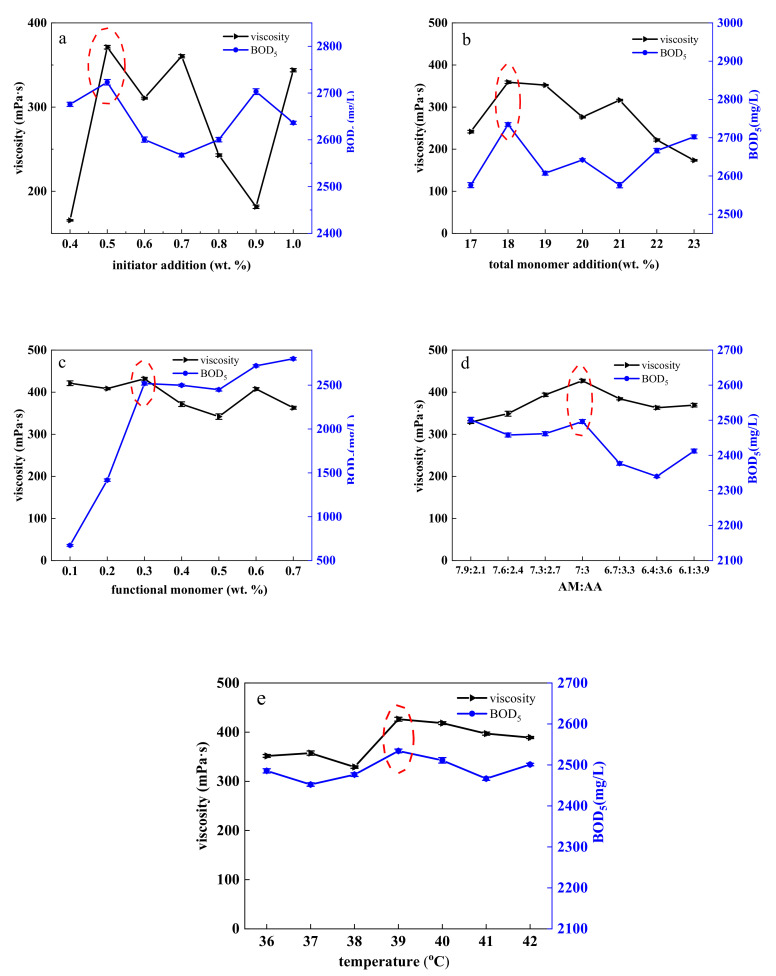
Optimal (**a**) initiator addition, (**b**) total monomer addition, (**c**) functional monomer, (**d**) AM:AA (acrylic acid: acrylamide) and (**e**) temperature of HPDACS.

The effects of initiator addition, total monomer concentration, functional monomer addition, AM:AA (mass ratio), and reaction temperature on polymer viscosity and BOD_5_ were investigated by single-factor experiment, and the results are shown in Figure 4. The following optimal synthesis conditions were established: initiator addition of 0.5 wt.%, total monomer concentration of 18 wt.%, functional monomer addition of 0.3 wt.%, AM:AA of 7:3, and reaction temperature of 39 °C.

The range of various influencing factors was further narrowed through another orthogonal test, and the optimal level of HPDACS was finally determined as follows: AM:AA > functional monomer addition > total monomer concentration > reaction temperature > initiator addition. The optimal synthesis conditions of each factor were as follows: AM:AA of 6.9:3.1, amount of functional monomer of 0.26 wt.%, total monomer concentration of 17.6 wt.%, reaction temperature of 39 °C, and initiator addition of 0.46 wt.% (details of orthogonal test, single-factor test, and orthogonal test are given in the Supplementary Materials).

The best synthesis conditions of HPDACS were finally determined by the combination of orthogonal test, single-factor test, and orthogonal test. The subsequent HPAM and HPDA were synthesized under the same conditions as contrasts, and the specific copolymerization conditions of the polymers are shown in Table 3.

### 3.2. Characterization

#### 3.2.1. FT-IR Spectroscopy

**Figure 5:**
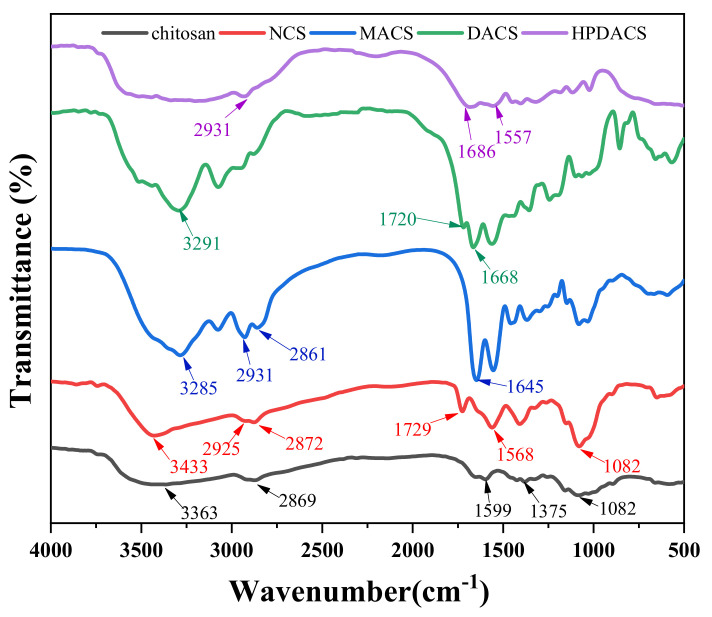
Infrared spectra of chitosan, NCS, MACS, DACS and HPDACS.

The chemical structures of chitosan, NCS, MACS, DACS, and HPDACS were confirmed by FT-IR spectroscopy, and the results are shown in Figure 5. According to the spectrum of chitosan, the peaks at 3363 and 3261 cm^–1^ represent the stretching vibrations of –OH and –NH_2_, respectively, which were greatly affected by H bonds. The absorptions at 2922, 2869, and 1082 cm^–1^ in chitosan indicate the existence of –CH_3_, –CH_2_, and –C–O–, respectively. When chitosan reacted with methyl acrylate, an ester group was introduced into the chitosan molecule. Compared with that of chitosan, the absorption peak at 1729 cm^–1^ in the spectrum of NCS is actually the characteristic absorption peak of –C = O–, indicating their successful reaction. According to the spectrum of MACS, except for the absorption peaks corresponding to NCS, the ester group absorption peak at 1729 cm^–1^ disappeared, and the characteristic absorption peak of –CONH– was found at 1645 cm^–1^. This finding indicated that the ester group in NCS reacted effectively with MA2.0. In the spectrum of DACS, the characteristic absorption peaks of –C = O and –C = C– were found at 1720 and 1668 cm^–1^ because of the introduction of the double bond and ester groups. This result reveals that the molecular structure designs are consistent with the actual results. The infrared spectrum of HPDACS showed a wide absorption band between 3500 and 3100 cm^–1^ assigned to the multimolecular association between carboxyl groups. The peak at 2931 cm^–1^ was the stretching vibration of –CH_2_, and the peak at 1686 cm^–1^ was attributed to –CONH_2_. In-plane bending vibration of –NH– was presented at 1557 cm^–1^. These results confirm the hyperbranched structure of HPDACS.

#### 3.2.2. X-ray Diffraction

**Figure 6:**
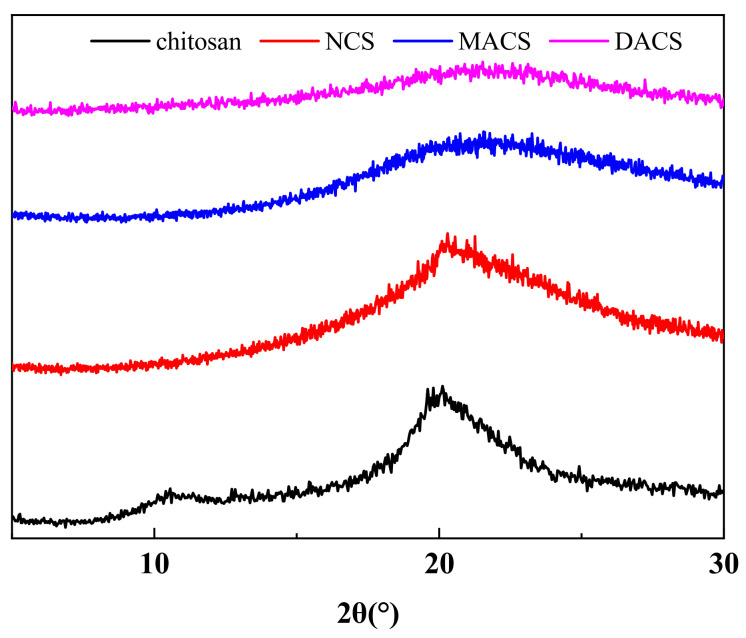
X-ray diffraction patterns of chitosan, NCS, MACS and DACS.

Figure 6 shows the X-ray diffraction pattern of chitosan, NCS, MACS, and DACS. Characteristic diffraction peaks of chitosan were observed at 2θ = 10° and 2θ = 20°. From NCS to DACS, the characteristic diffraction peak of 2θ = 10° disappeared, and the characteristic diffraction peak of 2θ = 20° gradually weakened. This phenomenon is mainly due to the continuous destruction of intermolecular and intramolecular hydrogen bonds on the chitosan surface with the progress of the reaction, resulting in the weakening and disappearance of the diffraction peak. This result further proves the reasonability of the molecular structure design.

#### 3.2.3. C and N Elemental Analysis

The mass percentage contents of the C and N elements of NCS, MACS, and DACS are shown in Table 4. The theoretically calculated values of C and N elements in NCS, MACS, and DACS were not substantially different from the actual measured values. Hence, the modified product could be determined as the expected target product, and the molecular structure of monomers in each step could be determined.

#### 3.2.4. ^1^HNMR

**Figure 7:**
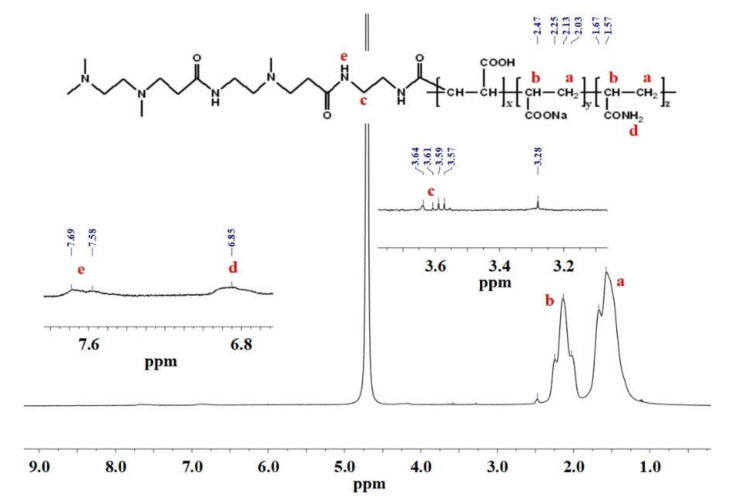
Hydrogen nuclear magnetic resonance (^1^HNMR) spectrum of HPDACS.

Figure 7 shows the ^1^HNMR spectrum of HPDACS. The chemical shifts at 1.57 and 2.2 ppm were related to the proton of –CH_2_ on AA and AM chains, respectively. The signal-observed peak from 3.5 ppm to 3.7 ppm was caused by the proton of –CH_2_–NH–, indicating the existence of the branched monomer DACS in HPDACS. The peak values at 6.85 and 7.69 ppm were attributed to the proton of –NH– on CS and the proton of –NH–CO– on the branched monomer chain, respectively. Hence, the structure of HPDACS was consistent with the original assumption based on ^1^HNMR results.

#### 3.2.5. Molecular Weight

Static light scattering was used to measure the scattered light intensity data of polymer solution at multiple angles and concentrations. The scattered light intensity values at zero angle and concentration were obtained through extrapolation fitting, and the weight-average molecular weight and other parameters are calculated by the light scattering formula.

Berry plot [41] was adopted for extrapolation and calculated according to Equation (2):

(2) $$\sqrt{\frac{Kc}{R_{\theta }}}=\sqrt{\frac{1}{M_{w}}+\frac{16\pi^{2}}{3\lambda^{2}}\frac{1}{M_{w}}(R_{g}){}^{2}\sin^{2}\left(\frac{\theta }{2}\right)}$$

where $K=\frac{4\pi^{2}}{N\lambda_{0}}n^{2}{\left(\frac{\partial n}{\partial c}\right)}^{2}$, *N* is Avogadro’s constant, *n* is the refractive index of the solution, *c* is the solution concentration, *M_w_* is weight-average molecular weight, *R_g_* is gyration radius of a polymer, *θ* is the scattering Angle, Rayleigh ratio $R_{\theta }=r^{2}\frac{I_{\theta }}{I_{0}}$, *r* is the distance from the detection point to the scatterer, *I_0_* is the incident light intensity, and *I_θ_* is the scattered light intensity.

**Figure 8:**
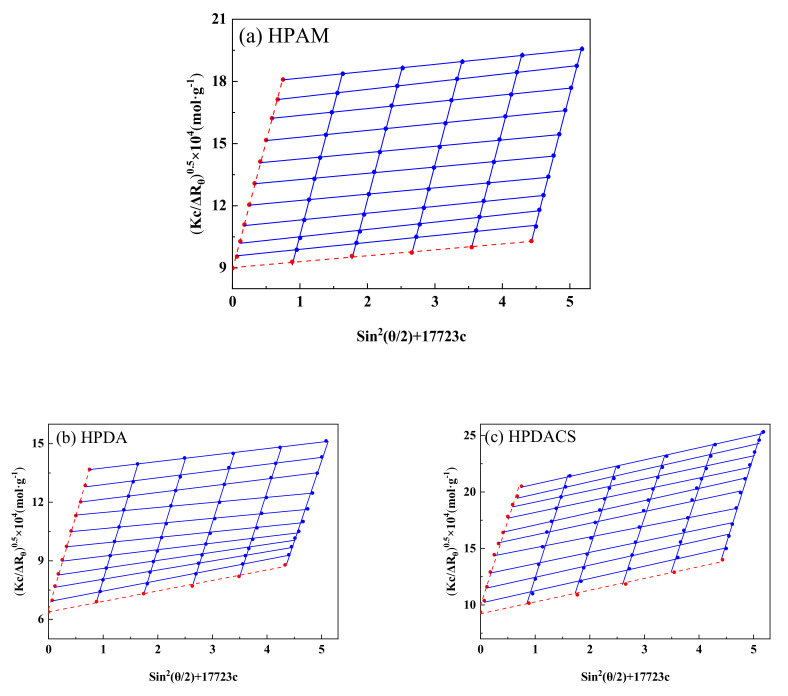
Berry curves of (**a**) HPAM (partially hydrolyzed polyacrylamide), (**b**) HPDA (dendritic polymer) and (**c**) HPDACS.

The fitting graph in Berry form is nonlinear. After deformation, the intercept of the vertical axis is *M*_w_^−1/2^ when *sin*^2^ (*θ*/2) + 17723c is the abscissa and (*Kc*/Δ*R_θ_*)^0.5^ is the ordinate. The ratio between the intercept and the longitudinal axis data is relatively large, and the error of experimental data has minimal influence on the determination of weight-average molecular weight. Therefore, the Berry algorithm was used for mapping to calculate the corresponding polymer solution weight-average molecular weight (*M*w). Finally, the weight-average molecular weights of HPAM, HPDA, and HPDACS were converted to 7.69 × 10^6^, 7.31 × 10^6^, and 8.52 × 10^6^ g/mol, respectively. The results are shown in Figure 8.

#### 3.2.6. Hydrodynamic Radius

**Figure 9:**
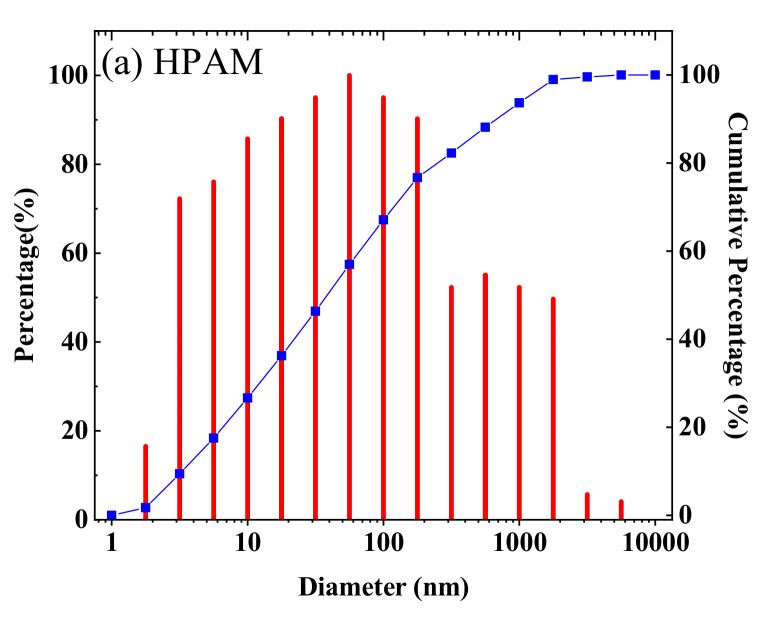

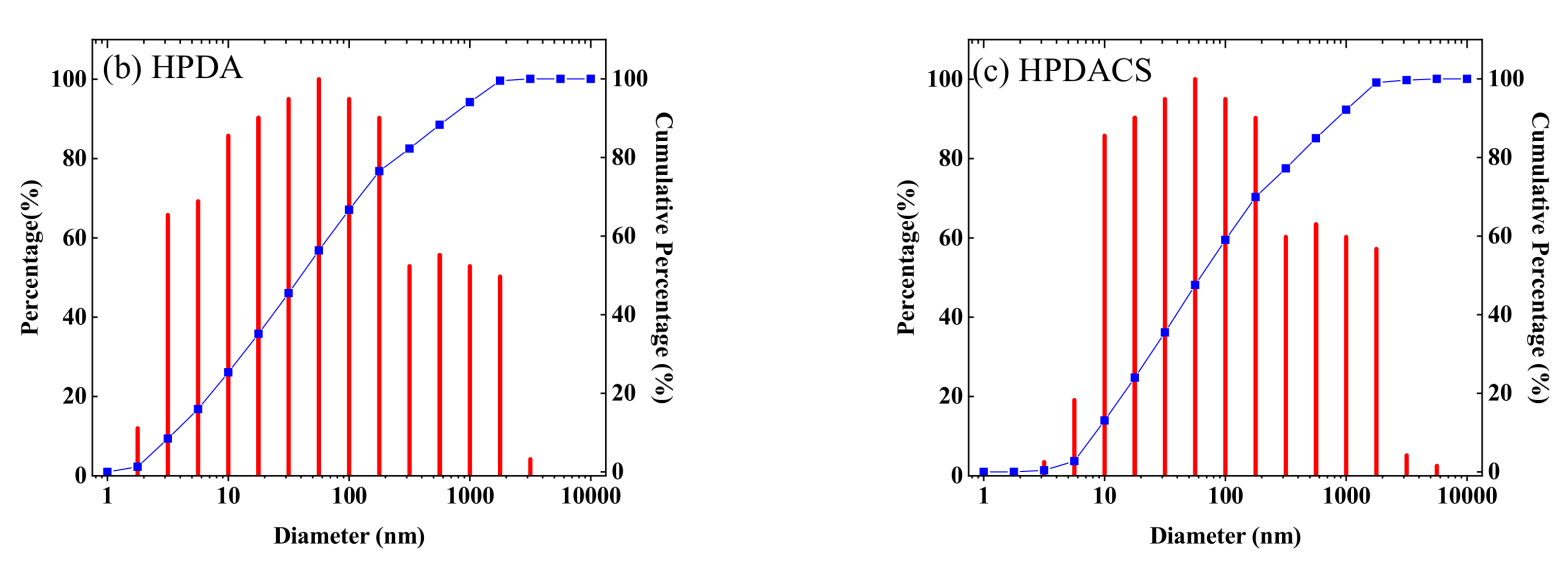
Diameter distribution of (**a**) HPAM, (**b**) HPDA and (**c**) HPDACS.

Figure 9 shows that the hydrodynamic radius of HPAM was 142 nm, which was larger than that of HPDA at 117 nm but smaller than that of HPDACS at 185 nm. These differences occurred because HPDA has a branched structure due to the introduction of branched monomer, and its molecular chain has many angles to extend outward—consistent with the original assumption. Hence, its hydrodynamic radius decreases. Although HPDACS has hyperbranched structure, the modified chitosan functional monomer as the core also occupies a certain space and thus, increases the hydrodynamic radius of the material.

#### 3.2.7. Morphology

**Figure 10:**
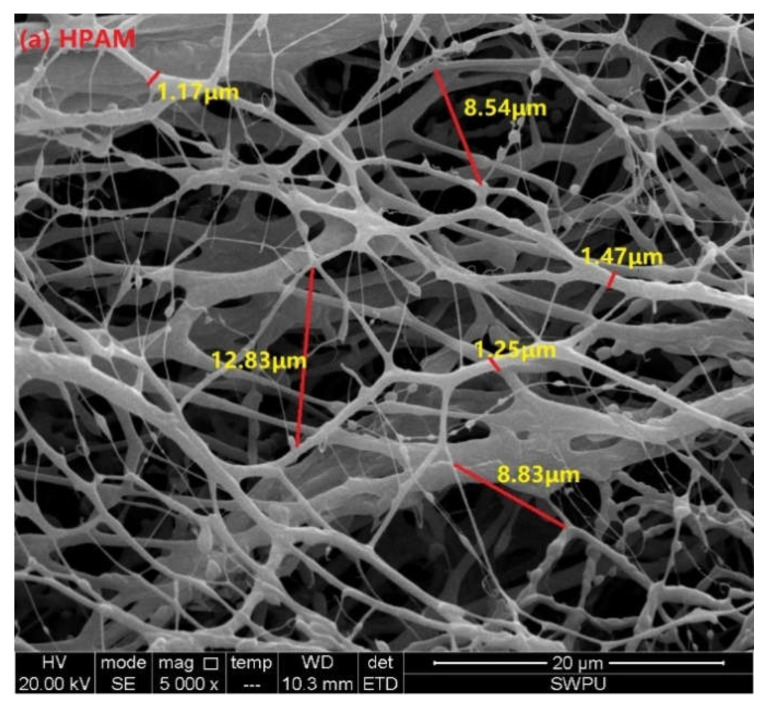

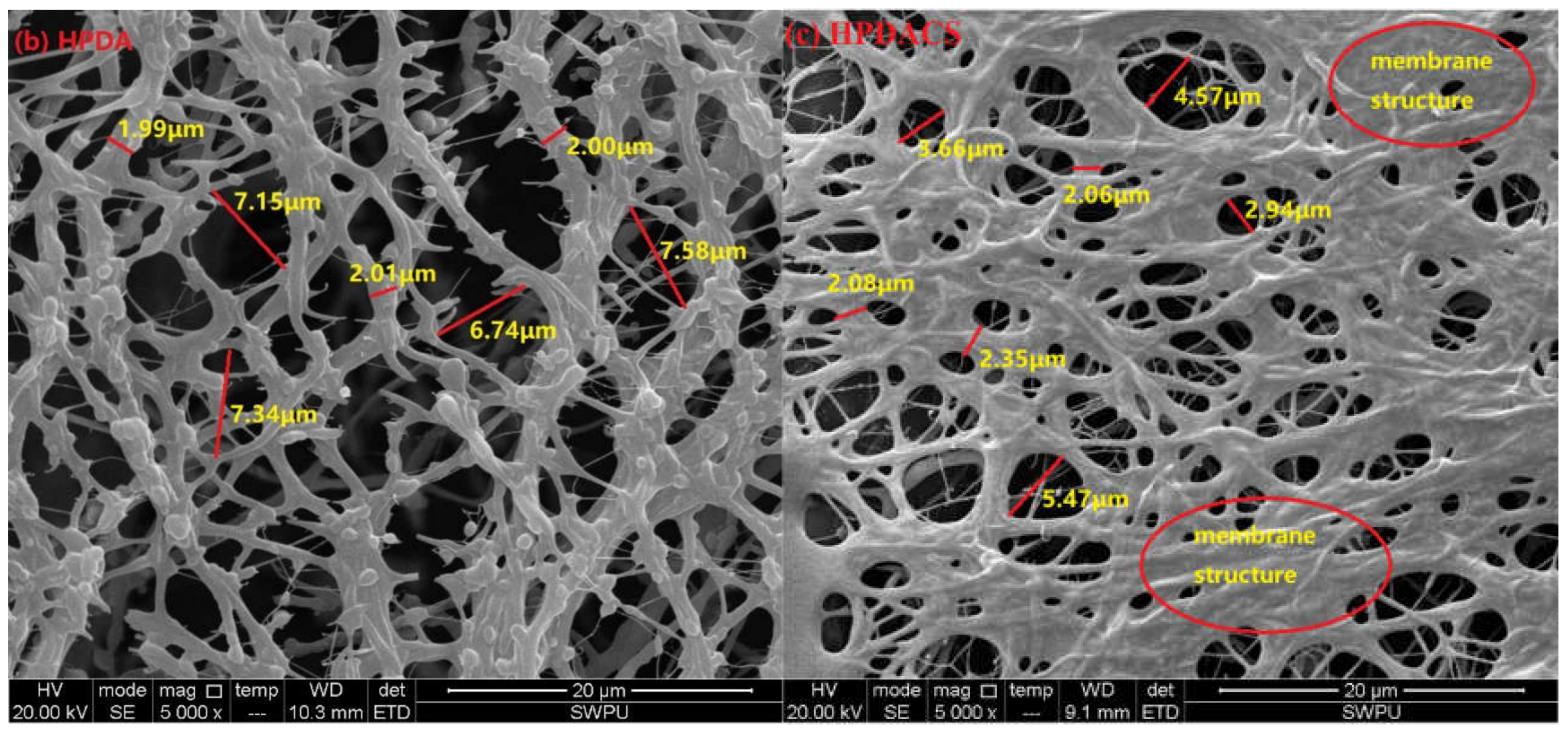
Environmental scanning electron microscopy (ESEM) morphologies of (**a**) HPAM, (**b**) HPDA and (**c**) HPDACS.

An ESEM system with an accuracy of 20 μm and magnification of 5000× was used to investigate the morphologies of HPAM, HPDA, and HPDACS in ultrapure water (Figure 10). As shown in Figure 10a, the structure of HPAM molecule is relatively loose, and its compactness is poor. The molecular skeleton is thin, and its size is approximately 1.1–1.5 μm. The molecular coil groups of HPAM exhibited weak entanglement and formed a certain wiredrawing network structure, and large holes were generated in the grid with 8–13 μm size, such a loose structure will reduce the strength of the polymer solution. Figure 10b shows that compared with that of HPAM, the skeleton of HPDA is thicker (the average size is approximately 2 μm), the network structure is relatively dense, and the cavity structure is smaller. This phenomenon occurred because the introduced branched monomer forms a certain strength of branched structure, and the molecular chain is tightly wound. The structure of HPDACS (Figure 10c) is different from those of HPAM and HPDA: the framework connecting the aggregates is thicker (approximately 2.1–2.4 μm), the network structure is more compact, the cavity of physical network structure is more uniform, and the membrane formed is more conducive to improve the structure strength of HPDACS. This result is mainly because the modified chitosan monomer and branched monomer in HPDACS formed a dense and structured dendritic skeleton, and the chitosan structure enhances the rigidity of polymer molecular chains to a certain extent, forms some membranes (no similar membrane structure was found in HPAM and HPDA) and improves the temperature and shear resistance of the polymer to a certain extent.

### 3.3. Solution Properties

#### 3.3.1. Solubility

**Figure 11:**
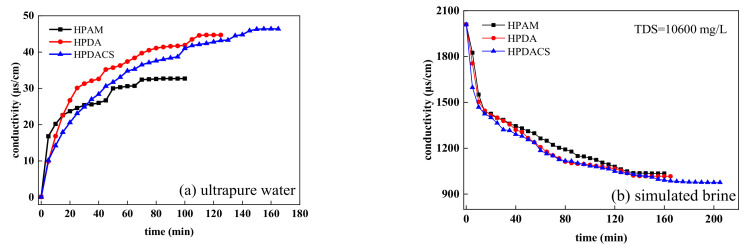
Relationship between polymer dissolution time and conductivity in (**a**) ultrapure water and (**b**) simulated brine.

The relationship curves between conductivity and dissolution time of HPAM, HPDA, and HPDACS in ultrapure water and simulated brine are shown in Figure 11. The dissolution process of the three polymers all conformed to the general law of polymer dissolution, that is, first swelling and then dissolving. As shown in Figure 11a, HPAM with linear structure had the shortest dissolution time at 75 min, which was twice as long for HPDACS with hyperbranched structure. On the one hand, the higher weight-average molecular weight of HPDACS compared with that of HPAM resulted its longer dissolution time. On the other hand, the dissolution time of HPDACS was delayed because its network structure was dense and intertwined closely.

Figure 11b shows after the polymer was dissolved in the simulated brine, the ions were shielded and weakened by the charge, resulting in a negatively increased conductivity value for the solution. Compared with that in ultrapure water, the time required for the complete dissolution of the three polymers in simulated brine was longer. This phenomenon occurred possibly because the inorganic ions in the solution compress the double electron layers of the swelling hydration film and thus increase the dissolution time of the polymer. Polymers with high weight-average and complex structure require a long time for complete dissolution.

#### 3.3.2. Thickening Ability

**Figure 12:**
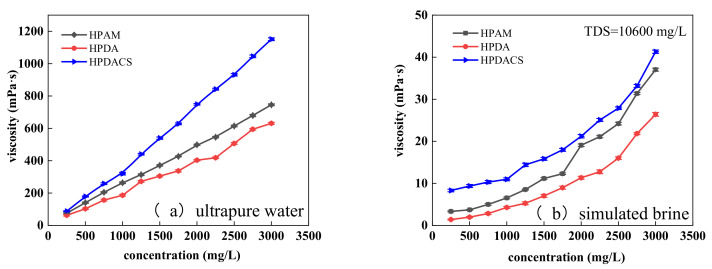
Effect of concentration on polymer viscosity in (**a**) ultrapure water and (**b**) simulated brine.

Figure 12a shows that the viscosity of HPAM, HPDA, and HPDACS polymers gradually increased with the concentration of the polymer solution. In the dilution zone, the polymer in the solution existed in the form of single molecule, and the viscosity increased slowly. With the increase in concentration, polymer molecules intertwined and even formed a network structure. In addition, the internal friction of molecular motion increased, resulting in an increased flow resistance and thus causing a sharp increase in the viscosity of polymer solution. When the concentration of the polymer solution prepared by ultrapure water was 250 mg/L, the viscosity of the three polymers was not different. When the concentration of polymer solution increased to 1500 mg/L, the viscosity of HPDACS reached 539.2 mPa·s, which was substantially higher than those of HPAM at 373.3 mPa·s and HPDA at 304.7 mPa·s, indicating the excellent thickening ability of HPDACS. The hyperbranched structure of HPDACS increases the chance of entanglement between molecular groups, and the shortened distance between molecular chains increases the intermolecular force and association effect. Meanwhile, the introduction of chitosan rigid structure enhances the viscosity enhancement of the polymer.

Figure 12b shows that given the effects of inorganic ions, the viscosity of the three polymer solutions at the same concentration were remarkably lower in brine than in ultrapure water. Under the same concentration conditions, HPDACS had higher viscosity than HPAM and HPDA and showed better viscosity-increasing ability in simulated brine. When the concentration of polymer solution was 250 mg/L, the viscosity of HPDACS was 8.3 mPa·s, which was higher than the 3.4 mPa·s of HPAM and 1.5 mPa·s of HPDA. When the polymer concentration increased to 3000 mg/L, the viscosity of HPDACS was 41.3 mPa·s, which was higher than the 37 mPa·s of HPAM and 26.3 mPa·s of HPDA. This phenomenon occurred because the addition of small molecular electrolytes in the system changes the polarity of the solution and shields the electrostatic attraction within the molecules. In addition, by pressing on the double electrical layer of the polymer chain, the inorganic ions force the polymer chain to curl, reduce the size of the polymer coil, and weaken the thickening ability of the polymer.

#### 3.3.3. Temperature Resistance

**Figure 13:**
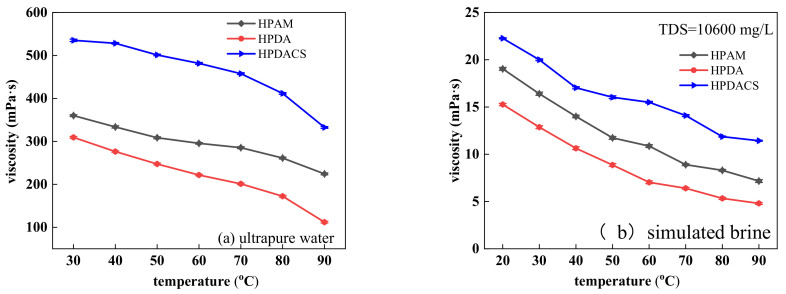
Effect of temperature on polymer viscosity in (**a**) ultrapure water and (**b**) simulated brine.

With successive increases in temperature, the viscosity of polymers was further lowered. On the one hand, when the temperature rises, the thermal movement of water molecules in the hydration layer film increases, and the chemical bonds on the molecular chains are easily broken. In addition, the internal rotation of single bonds in the molecule is increased, causing the molecular chain to curl and the corresponding hydrodynamic radius to decrease. On the other hand, polymers are prone to hydrolysis at high temperatures, leading to a decrease in polymer viscosity. When the solution temperature increased to 90 °C in ultrapure water, the viscosity retention rate of HPDACS was 62%, which was higher than that of HPAM at 59% and HPDA at 36%. This finding is mainly due to the hyperbranched structure of HPDACS. The introduction of chitosan structure into the polymer molecular structure increases the internal friction between molecules, thus slowly decreasing the viscosity of HPDACS and giving HPDACS a good temperature resistance. The results are shown in Figure 13a.

The viscosity of all three polymers in brine decreased to some extent compared with that in ultrapure water. By pressing on the double electrical layer of polymer chain, inorganic ions force the molecular chain of polymer to curl, resulting in the decrease in polymer size, hydrodynamic radius, temperature resistance. Figure 13b shows that when the temperature reached 90 °C in simulated brine, the viscosity retention rates of HPAM, HPDA, and HPDACS were 37.9%, 32.2%, and 51.8%, respectively. This finding indicated that HPDACS showed the best temperature resistance among the three polymers.

#### 3.3.4. Salt Resistance

In an actual reservoir environment, the salinity of formation water greatly influences the properties of polymer solution. In most cases, the polymer solution viscosity decreases with the increase in salinity, thus influencing the displacement effect. In this study, Na^+^, Ca^2+^, and Mg^2+^ were considered the main effects of inorganic ions on the polymer in water formation.

**Figure 14:**
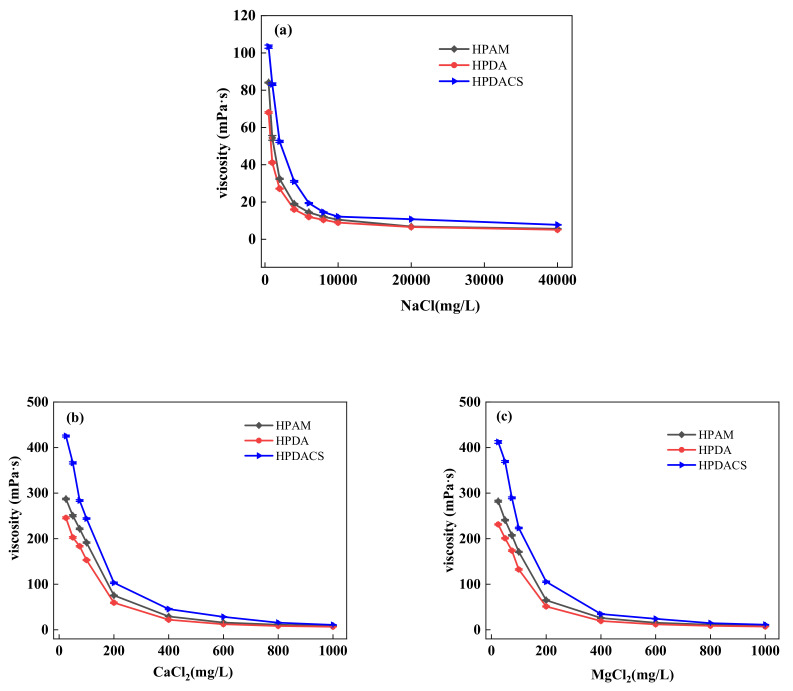
(**a**) NaCl; (**b**) CaCl_2_ and MgCl_2_ effects on polymer viscosity.

Figure 14 shows that with the increase in salt concentration, the viscosity of the three polymers decreased substantially and finally stabilized. When the salt concentration increases, the cation and the anion group on the polymer molecular chain form a counter-ion pair, which has a shielding effect on the negative charge on the polymer chain. This phenomenon induces a dehydration effect, which weakens the electrostatic repulsion between the anion groups. In the presence of inorganic ions, the polymer molecular chain curls severely and reduces the hydrodynamic radius of the molecule and the viscosity of the solution. Given that the divalent salt has a large charge numbers, the electric double layer is further compressed with the increase in charge numbers, resulting in the intensified curling degree of polymer molecular chains and the increased range of solution viscosity reduction.

Under the same conditions, HPDACS had a higher viscosity retention rate than HPAM and HPDA because the modified chitosan monomer and branched monomer make the HPDACS network structure highly compact and regular, thereby potentially increasing the crimping difficulty of HPDACS and reducing the crimping degree of its molecular chain to a certain extent. Moreover, the steric hindrance effect of chitosan structure also weakened the influence of metal ions on polymer molecular chains. Hence, HPDACS show better salt resistance than the two. When the added amount of salt in the solution reached a certain amount, the shielding effect of the metal ions was maximized, and the macromolecular chain could no longer be curled. Hence, the viscosity of the polymer solution did not change.

#### 3.3.5. Anti-Shearing Ability

The polymer solution was subjected to a series of shear and tensile degradation during preparation and pumped through pipelines, wellbore, perforated holes, and porous media, resulting in their decreased viscosity, increased water–oil mobility ratio, and poor displacement effect [42]. The viscosity changes of HPAM, HPDA, and HPDACS before and after shearing under different water qualities (ultrapure water and simulated brine) and at different standing times after shearing (0, 12, and 24 h) are shown in Table 5 and Table 6.

Table 5 shows that in ultrapure water, the viscosity retention of HPAM, HPDA, and HPDACS decreased with the increasing shear rate. In particular, the viscosity retention rate of HPDACS was higher than that of HPAM and HPDA, indicating the good shear resistance of the former. This result occurred because the introduced branched monomers provided hyperbranched network structures for HPDA and HPDACS. After cutting by a Waring mixer, only part of the branched chains was cut off, and the main chains did not change remarkably. The weight-average molecular weight and hydrodynamic volume were not seriously affected, and the viscosity of the solution of HPDA and HPDACS were therefore relatively less affected by shear action than that of HPAM. When the linear polymer HPAM was subjected to mechanical shear, part of the polymer main chain was broken, and the molecular size was reduced. Hence, the viscosity retention of HPAM was lower than those of HPDA and HPDACS. Compared with HPDA, HPDACS had a higher viscosity due to the rigid structure of chitosan. By contrast, the synergistic effect of chitosan and branched monomer provided a compact and regular branch network structure for HPDACS. When the shear rate changed from 3500 r/min to 11,500 r/min, its viscosity retention rate decreased by 11.22%, which is better than that of HPDA that dropped by 16.47%. This finding indicated that the introduction of chitosan-modified hyperbranched monomer increased the shear resistance of the polymer. These results are shown in Table 5.

Compared with those in ultrapure water, the viscosity and viscosity retention of the three polymers prepared in simulated brine all decreased to a certain extent because the inorganic ions in the solution greatly influence the structure of the polymer, directly act on the electric double layer of the polymer chain, and reduce the coil size of the polymer and the regularity of its network structure. After shearing, the viscosity retention rate was decreased to a large extent. These results are shown in Table 6.

#### 3.3.6. Anti-Aging Ability

A polymer solution moving in porous media is affected by the environment and suffers from different degrees of high-temperature degradation and shear tensile degradation, thus affecting the viscosity of the polymer solution. Therefore, the polymer aging resistance directly affects the effective time of polymer-flooding operations.

The anti-aging abilities of the three polymers are shown in Table 7. HPAM, HPDA, and HPDACS all showed certain antiaging abilities under different water quality conditions. The viscosity of the three polymers decreased rapidly within 1–30 days, and the rate of viscosity decrease gradually slowed down after 30 days. In ultrapure water, the viscosity of 1500 mg/L HPDACS solution remained at 442.1 mPa·s after aging at 60 °C for 90 days, and the viscosity retention rate was 81.99%, which was better than that of HPAM at 79.00% and HPDA at 74.99%. After aging at 60 °C for 90 days in simulated saline water, the viscosity retention rate of HPDACS was slightly lower than that in ultrapure water at 79.49% (better than that of HPAM at 70.91% and HPDA at 61.43%), indicating that HPDACS has a good antiaging ability that meets the performance requirements for polymer flooding.

#### 3.3.7. Rheology

**Figure 15:**
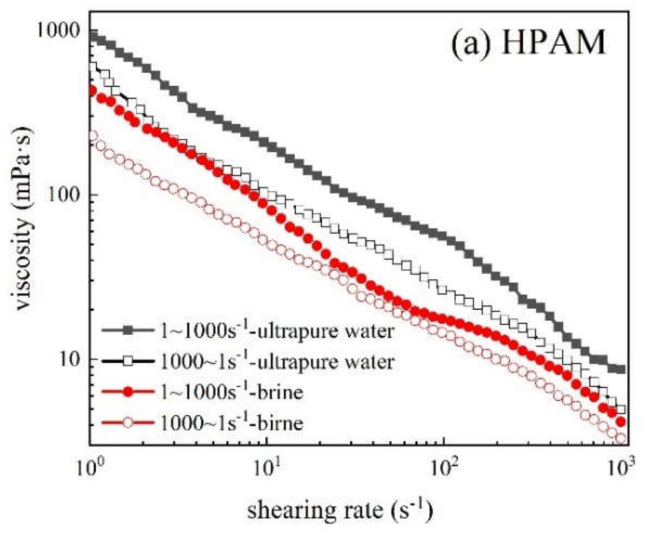

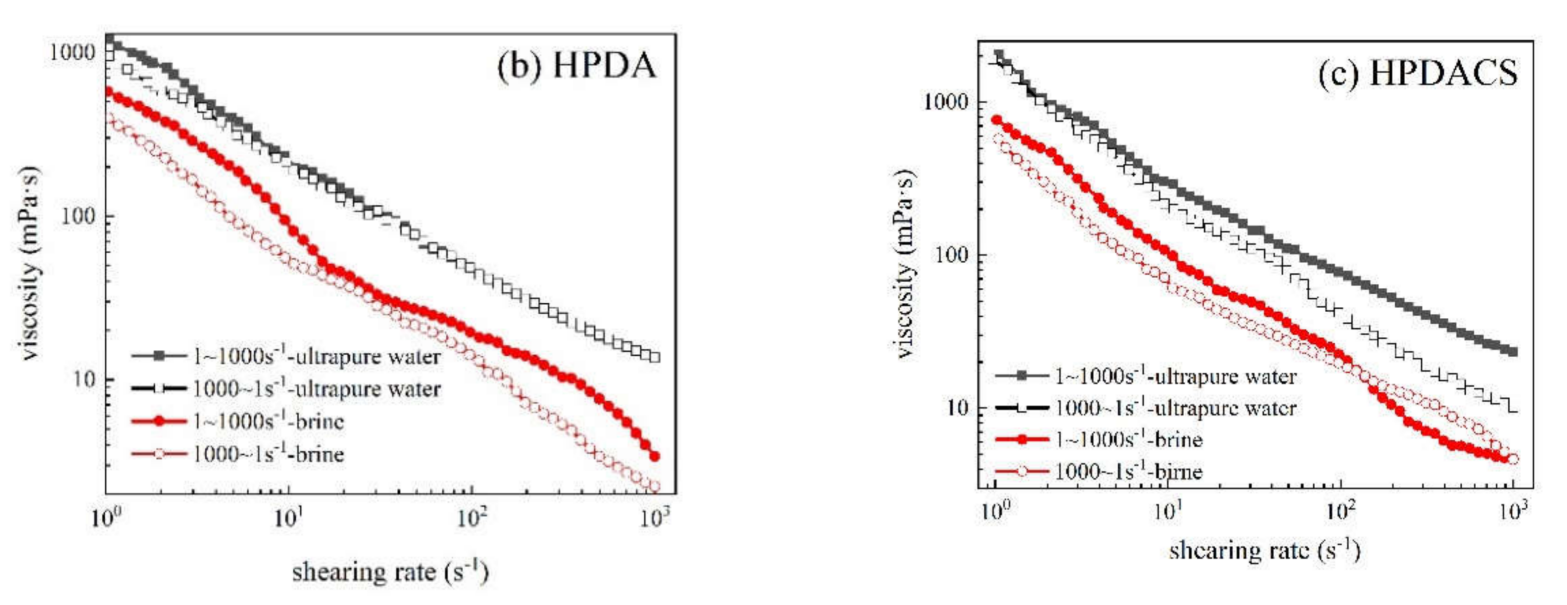
Shear-thinning behaviors of (**a**) HPAM, (**b**) HPDA and (**c**) HPDACS.

In ultrapure water and simulated brine, the rheological curves of three polymers (1500 mg/L) at 60 °C and shear rate of 1–1000 s^−1^ are shown in Figure 15. Within the shear rate curve of 1–1000 s^−1^, the apparent viscosity of the three polymer solutions decreased gradually with the increase in shear rate, and all showed the characteristics of pseudoplastic fluid that is conducive to improve the injectability of polymer solution. This phenomenon occurred due to the intermolecular entanglement and interaction in the polymer solution system. When the polymer solution was continuously subjected to shear action, the entangled structure was destroyed and cannot be restored to the original state in time. Therefore, the polymer solution showed decreased viscosity and shear dilution. When the shear rate was 1–1000 s^−1^, the viscosity of HPDACS was higher than those of HPDA and HPAM in ultrapure water and brine. The viscosity retention rate of HPDACS was also higher than those of HPAM and HPDA from high shear to low shear (1000–1 s^−1^).

The rheological behavior of polymer solution was studied by the Power Law model to describe the variation of apparent viscosity of polymer solution with shear rate. The value of the power law exponent n reflects the degree of deviation of rheological properties of fluids from those of Newtonian fluids. Consistency coefficient K is a measure of viscosity and thus can reflect the sweep efficiency of polymer solution in porous media. The power law formula [43] is as follows:

(3) $$\eta \left(\gamma \right)=K\gamma^{n-1}$$

where η is the viscosity of polymer, Pa·s, K is the consistency coefficient, Pa·s^n−1^, *γ* is the shear rate, s^−1^, *n* is the power law index, dimensionless.

A small n indicates the strong pseudoplasticity of polymer solution and the intense stretching of polymer molecules in aqueous solution. A high K value indicates the great non-Newtonian characteristics of the fluid and the strong thickening ability of the solution. The power law parameters of the three polymers in ultrapure water and brine are shown in Table 8.

Table 8 shows that the K value of HPDACS is the largest, and its n value is the minimum in ultrapure water and simulated saline water. Therefore, HPDACS solution has strong non-Newtonian property and pseudoplasticity. After experiencing low shear to high shear to low shear (1–1000–1 s^−1^), HPDACS exhibited the lowest K values ((K_1_-K_2_)/K_1_) of 6.45% (in ultrapure water) and 28.26% (in simulated brine), which were lower than the 39.03% (in ultrapure water) and 51.02% (in simulated brine) of HPAM and 25.66% (in ultrapure water) and 34.04% (in simulated brine) of HPDA. When the shear rate was low, the molecular chains of the polymer were oriented and stretched in the flow. When the shear rate increased, the molecular chain of the polymer was partially broken, and some polymer molecular association structures were destroyed; hence, the viscosity of the polymer decreased.

Owing to the linear structure of HPAM, the weak association between molecules is easily destroyed by shear stress, resulting in a large decrease in viscosity and corresponding consistency coefficient K value. When dendrimer was introduced into HPDA, its shear resistance was improved to a certain extent. Hence, the decrease in the rate of K value after shearing was smaller than that of HPAM. For HPDACS, only a small part of the branched chain broke at high shear rate because of its 3D hyperbranched structure with chitosan as the core. Hence, the decrease in the rate of consistency coefficient K was low, and its shear resistance was strong. Introducing chitosan provides HPDACS better shear resistance than HPDA. Under the same conditions, the consistency coefficients of the three polymers in brine were lower than those in ultrapure water because inorganic ions compress the electric double layer, thus increasing the curl degree of the polymer molecular chain and reducing the shear resistance and thickening ability of the polymer.

#### 3.3.8. Viscoelasticity

**Figure 16:**
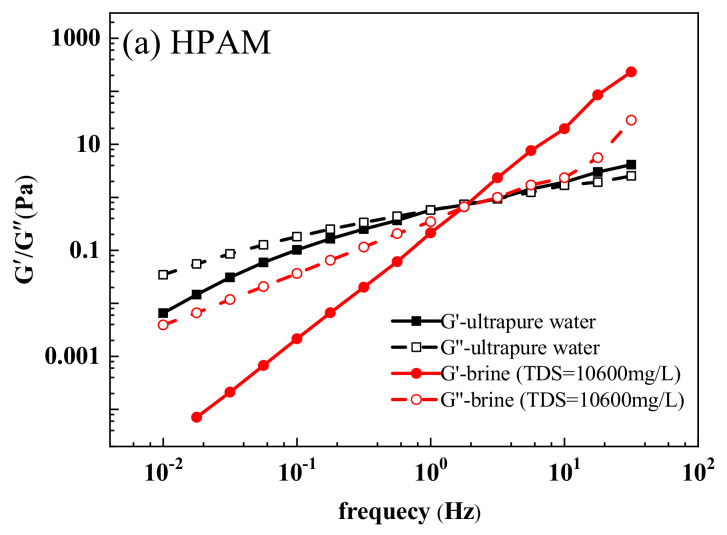

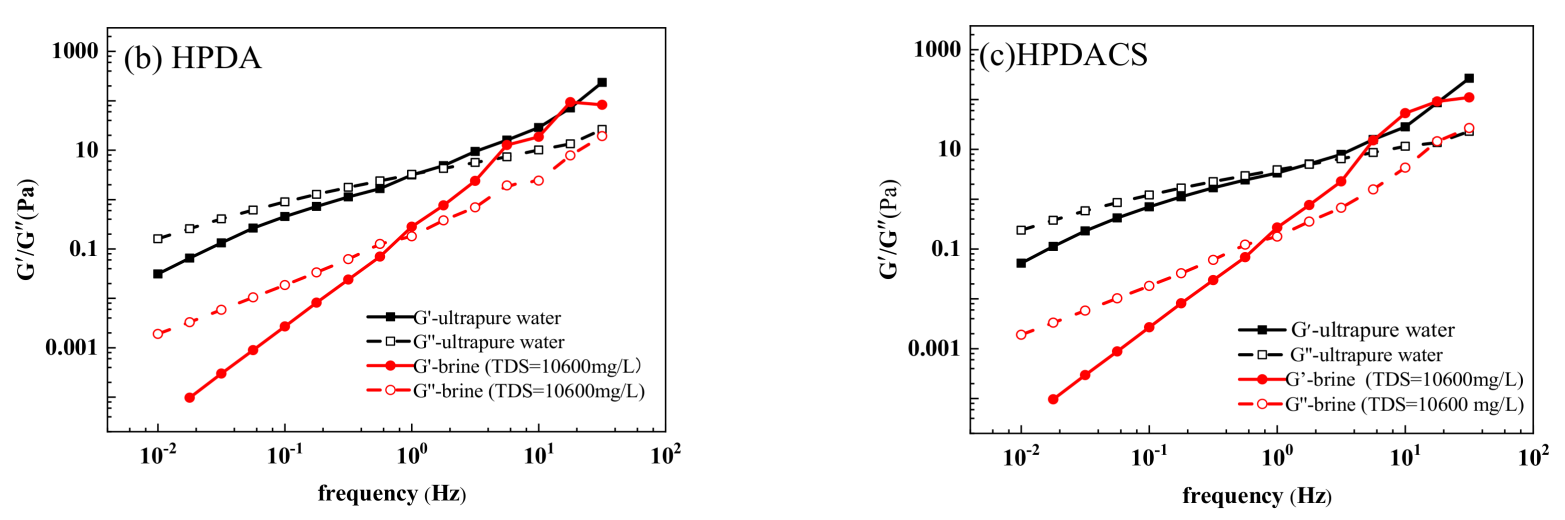
Viscoelastic behaviors of (**a**) HPAM, (**b**) HPDA, and (**c**) HPDACS.

The elastic modulus and viscosity modulus of HPAM, HPDA, and HPDACS in ultrapure water and simulated saline water are shown in Figure 16. In the scanning frequency range, the elastic modulus and viscosity modulus of the three polymers increased with the scanning frequency, and their *G*′ and *G*″ had an intersection point. When the frequency was lower than the intersection point, the polymer solution was dominated by viscosity, which allows the solution to establish appropriate flow resistance in porous media. When the frequency was greater than the intersection value, the polymer solution was mainly elastic, which is helpful for polymer deformation and migration in porous media and the striping of residual oil. The intersection of *G*′ and *G*” is defined as the crossing modulus (*G*c), which quantitatively characterizes the viscoelastic properties of polymers. The reciprocal of frequency corresponding to *G*c is the relaxation time *λ*c of polymer solution, and *λ*c indicates the disassociated time of the polymer and thus can be used to describe the viscoelasticity of the polymer [44]. Under the same conditions, *G*′ and *G*” of the three polymers were HPDACS > HPDA > HPAM, indicating the good viscoelasticity of HPDACS. Therefore, HDPACS has great potential in flooding displacement. The *G*c and *λ*c of the three polymers in ultrapure water and simulated saline water are shown in Table 9.

### 3.4. EOR Ability

**Figure 17:**
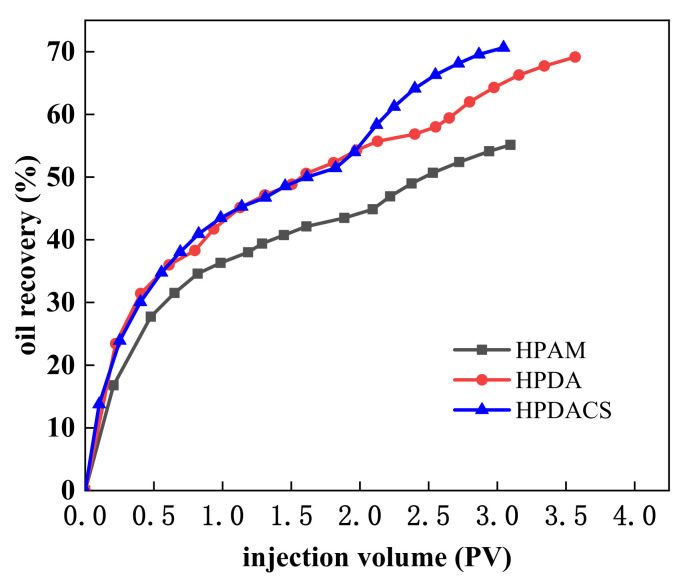
Enhanced oil recovery (EOR) abilities for HPAM, HPDA, and HPDACS.

The oil recovery of HPAM, HPDA, and HPDACS is shown in Figure 17 and Table 10. When the water content reached 95% and 0.3 PV HPAM was injected, HPAM flooding improved the crude oil recovery ratio by 10.65%, and the cumulative recovery ratio of the whole water flooding and HPAM flooding was 55.14%. Under the same conditions, the enhanced crude oil recovery ratio by HPDA flooding was 13.72%, and the cumulative recovery ratio by water flooding and HPDA flooding was 70.57%. When 0.3 PV HPDACS was injected, HPDACS flooding improved the crude oil recovery by 19.20%, which was higher than those by HPAM and HPDA, and the cumulative recovery was 70.65%.

The analysis shows that HPAM formed a single adsorption layer on the rock surface in the one-dimensional sandpack model, and the adsorption retention was relatively small. In the subsequent water flooding, HPAM was continuously scoured, resulting in limited sweep expansion capacity and a low recovery ratio. Compared with HPAM, HPDA with hyperbranched structure had more contact points with the rock surface and formed multilayer adsorption on the rock surface. Thus, the former can more effectively reduce the permeability of the water phase, establish higher residual resistance factor in porous media, and achieve higher oil recovery ratio than HPAM. Compared with HPAM and HPDA, HPDACS had a higher viscosity and denser branch network structure, which are helpful in establishing a high seepage resistance and improving water–oil mobility ratio during flooding displacement. Even in extended water flooding, HPDACS can still stick on the rock surface depending on its structural characteristics, thus expanding the sweep volume and improving the oil recovery ratio.

## 4. Conclusions

(1) Combined single-factor and orthogonal experiments revealed that the optimal synthesis conditions of HPDACS were an AM:AA of 6.9:3.1, a functional monomer addition of 0.26 wt.%, a total monomer concentration of 17.6 wt.%, a temperature of 39 °C, and an initiator of 0.46 wt.%. FT-IR and ^1^HNMR characterization confirmed the successful synthesis of HPDACS.

(2) The solution properties of HPDACS, including solubility, thickening ability, temperature and salt resistance, and aging resistance, were studied in ultrapure water and simulated brine. The results showed that HPDACS has a good thickening ability and temperature and salt resistance. After 90 days of aging, the viscosity retention rate of HPDACS exceeded 79.49%, which can meet the performance requirements of polymer flooding.

(3) The effects of mechanical shear rate on the viscosity of HPAM, HPDA, and HPDACS solutions were studied. When the shear rate increased, the viscosity retention of polymers decreased to varying degrees, but that of HPDACS in ultrapure water and simulated brine was higher than those of HPAM and HPDA, indicating its excellent shear resistance. Rheological experiments proved that HPDACS is a typical power law fluid and has good viscoelasticity.

(4) When the one-dimensional sandpack model had a permeability of 300 × 10^–3^ µm^2^, a polymer concentration of 1500 mg/L, and an injection volume of 0.3 PV, HPDACS effectively improved the oil recovery by increasing the recovery factor by 19.20%, which was higher than those of HPAM at 10.65% and HPDA at 13.72%. This finding indicates its great potential for oil displacement.

## Figures and Tables

**Table 1:** Ionic composition of the brine.

Ion	Na^+^	Ca^2+^	Mg^2+^	Cl^–^	TDS
Content (mg/L)	5000	100	100	5400	10,600

**Table 2:** Basic parameters of one-dimensional sandpack model of HPAM (partially hydrolyzed polyacrylamide), HPDA (dendritic polymer) and HPDACS (chitosan modified hyperbranched polymer).

Polymer	Concentration (mg/L)	Permeability (×10^–3^ μm^2^)	Porosity (%)	Oil saturation (%)
HPAM	1500	317	30.59	79.35
HPDA		346	30.77	85.19
HPDACS		327	30.63	82.49

**Table 3:** Copolymerization conditions of HPAM, HPDA, and HPDACS. AM:AA (acrylic acid: acrylamide).

Polymer	Total Monomer Concentration (wt.%)	AM:AA	Initiator (wt.%)	Functional Monomer (wt.%)	Temperature (°C)		pH		Reaction Time (h)
HPAM	17.6	6.9:3.1	0.46	-		39	7	6	
HPDA				0.26 (MA2.0)					
HPDACS				0.26 (DACS)					

**Table 4:** Mass percentage of C and N elements in NCS (modified chitosan), MACS (further modified chitosan), DACS (branched monomer modified chitosan).

Monomer	Element	Experimental Value (%)	Theoretical Value (%)
NCS	C	48.55	48.58
	N	0.056	0.057
MACS	C	51.85	51.86
	N	0.22	0.23
DACS	C	57.62	57.66
	N	0.16	0.16

**Table 5:** Viscosity changes of polymers prepared by ultrapure water before and after shearing.

Polymer	η_b_ (mPa·s)	Shear Rate (r/min)	η_a_ at Different Times (mPa·s)			Viscosity Retention (%)
			0 h	12 h	24 h	
HPAM	373.3	3500	307.1	308.7	308.7	82.69
		7000	278.8	280.4	282.0	75.54
		11,500	231.7	233.6	233.6	62.58
HPDA	304.7	3500	269.3	270.8	270.8	88.87
		7000	233.8	240.2	241.8	79.36
		11,500	214.2	220.6	220.6	72.40
HPDACS	539.2	3500	494.6	496.0	497.6	92.28
		7000	453.9	458.7	460.3	85.36
		11,500	429.2	435.5	437.1	81.06

Note: η_b_ is the polymer viscosity before shearing; η_a_ is the polymer viscosity after shearing; viscosity retention rate refers to the ratio of the viscosity of the polymer after shear to its initial viscosity, whose value is equal to η_a_/η_b_.

**Table 6:** Viscosity changes of polymers prepared by simulated brine before and after shearing.

Polymer	η_b_ (mPa·s)	Shear Rate (r/min)	η_a_ at Different Times (mPa·s)			Viscosity Retention (%)
			0 h	12 h	24 h	
HPAM	11.0	3500	5.2	5.3	5.3	48.18
		7000	4.1	4.2	4.3	39.09
		11,500	3.3	3.3	3.4	30.91
HPDA	7.0	3500	4.1	4.1	4.1	58.57
		7000	3.5	3.5	3.6	51.43
		11,500	2.8	2.9	3.0	42.86
HPDACS	15.6	3500	11.8	11.8	11.9	76.28
		7000	10.6	10.7	10.8	69.23
		11,500	9.4	9.5	9.5	60.90

Note: η_b_ is the polymer viscosity before shearing; η_a_ is the polymer viscosity after shearing; viscosity retention rate refers to the ratio of the viscosity of the polymer after shear to its initial viscosity, whose value is equal to η_a_/η_b_.

**Table 7:** Antiaging properties of polymers under different water quality conditions.

Aging Time (Days)	Viscosity (mPa·s)					
	Ultrapure Water			Simulated Brine		
	HPAM	HPDA	HPDACS	HPAM	HPDA	HPDACS
0	373.3	304.7	539.2	11.0	7.0	15.6
5	366.9	289.5	528.4	10.7	6.7	15.3
10	355.7	280.3	512.2	10.5	6.4	14.7
20	342.6	262.0	496.1	9.6	6.0	14.0
30	330.1	246.8	479.9	9.0	5.7	13.4
60	312.9	240.7	458.3	8.6	4.9	12.8
90	294.9	228.5	442.1	7.8	4.3	12.4

**Table 8:** The rheological parameters of HPAM, HPDA, and HPDACS.

Polymers	Shearing Rate (s^−1^)	Ultrapure Water			Simulated Brine		
		K_1_ (Pa·s^n−1^)	n	R^2^	K_2_ (Pa·s^n−1^)	n	R^2^
HPAM	1~1000	946.89916	0.31522	0.9969	436.8173	0.2905	0.9984
	1000~1	577.3057	0.2094	0.9853	213.9437	0.3909	0.9963
HPDA	1~1000	1276.2056	0.2744	0.9968	627.7289	0.2340	0.9919
	1000~1	948.7144	0.3462	0.9985	414.0435	0.1500	0.9970
HPDACS	1~1000	1945.5635	0.1772	0.9900	797.2804	0.1414	0.9961
	1000~1	1820.0343	0.0936	0.9976	571.9893	0.0377	0.9934

**Table 9:** *G*c (crossing modulus) and *λ*c (relaxation time) of HPAM, HPDA, and HPDACS.

Polymers	Ultrapure Water		Simulated Brine	
	*G*c (Pa)	*λ*c (s)	*G*c (Pa)	*λ*c (s)
HPAM	0.5903	1.0156	0.6478	0.5583
HPDA	3.1931	0.9916	0.1490	1.2192
HPDACS	4.8897	0.5583	0.1531	1.3071

**Table 10:** EOR (enhanced oil recovery) of HPAM, HPDA, and HPDACS.

Polymer	Permeability (×10^−3^μm^2^)	E_2_ (%)	E_1_ (%)	EOR (%)
HPAM	317	55.14	44.49	10.65
HPDA	346	70.57	56.85	13.72
HPDACS	327	70.65	51.45	19.20

## Supplementary Materials

The following are available online at https://www.mdpi.com/2073-4360/12/9/2130/s1, Figure S1: Infrared spectra of MA0.5, MA1.0, MA1.5 and MA2.0, Figure S2: ^1^HNMR of (a) MA0.5, (b) MA1.0, (c) MA1.5 and (d) MA2.0, Figure S3: Reaction scheme of NCS, Figure S4: Reaction scheme of MACS, Figure S5: Reaction scheme of DACS, Figure S6: Effect of initiator addition on polymer viscosity and BOD_5_, Figure S7: Effect of total monomer concentration on polymer viscosity and BOD_5_, Figure S8: Effect of functional monomer addition on polymer viscosity and BOD_5_, Figure S9: Effect of AM:AA on polymer viscosity and BOD_5_, Figure S10: Effect of reaction temperature on polymer viscosity and BOD_5_, Table S1: Optimal synthesis conditions of branched monomers, Table S2: Orthogonal test table of polymers, Table S3: Orthogonal test table of 4^5^ polymers.
