# Supplementary material for: Synthesis and Solution Properties of a Novel Hyperbranched Polymer Based on Chitosan for Enhanced Oil Recovery
Source: Polymers (Basel). 2020 Sep 18;12(9):2130. doi: 10.3390/polym12092130 (PMC7570182; doi:10.3390/polym12092130)
Supplement: Supplementary file 1 [file polymers-12-02130-s001.pdf]

Supplementary Materials

Synthesis and Solution Properties of a Novel Hyperbranched Polymer Based on Chitosan for Enhanced Oil Recovery

Qingyuan Chen [1,2], Zhongbin Ye [1,2,*], Lei Tang [1,2], Tao Wu [3], Qian Jiang [1,2] and Nanjun Lai [1,2,4,*]

1 School of Chemistry and Chemical Engineering of Southwest Petroleum University, Chengdu, Sichuan 610500, China; 201911000046@stu.swpu.edu.cn (Q.C.); 201811000045@stu.swpu.edu.cn (L.T.); 201921000222@stu.swpu.edu.cn (Q.J.)

2 Oil & Gas Field Applied Chemistry Key Laboratory of Sichuan Province, Chengdu, Sichuan 610500, China

3 Sanjiang Aerospace Jianghe Chemical Technology Co., Ltd., Yuan'an, Hubei 444200, China; 201821000247@stu.swpu.edu.cn

4 State Key Laboratory of Oil and Gas Reservoir Geology and Exploitation, Chengdu University of Technology, Chengdu, Sichuan 610059, China

* Correspondence: yezb@swpu.edu.cn (Z.Y.); lainanjun@swpu.edu.cn (N.L.); Tel.: +86-13094484238 (N.L.); Tel.: +86-13880551827 (Z.Y.)

1. Synthesis of branched monomers

Using ethylenediamine and methyl acrylate as raw materials, the final branched monomer (MA2.0) was synthesized repeatedly and iteratively through the Michael addition and Friedel-Crafts acylation reaction. The monomer synthesized in each step was followed by thin-layer chromatography (spot plate) to track the reaction process, and the product was separated and purified by vacuum distillation and column layer analysis. The specific steps were as follows:

(1) Under the protection of an ice bath and nitrogen, using dry methanol as the solvent, methyl acrylate solution was dripped into ethylenediamine solution using a constant-pressuredropping funnel at a rate of one to two drops per second. After the reaction was completed, placement was continued in the ice bath for 30 min, and then the reaction system was placed at 25 ℃, during which the reaction process was detected at all times through a spot plate. After the reaction was completed, the solvent methanol and excess methyl acrylate were removed by vacuum distillation at 45 ℃. The product was purified by column chromatography, with silica gel as the adsorbent and ethyl acetate:petroleum ether = 1:3 (volume ratio) as the eluent, and the eluent was detected by a spot plate at all times to determine the product. The eluent finally was removed by vacuum distillation to obtain a light yellow liquid (MA0.5) with a yield of 95.2%.

(2) Under the protection of an ice bath and nitrogen, using dry methanol as the solvent, ethylenediamine solution was dripped into MA0.5 solution with a constant-pressure-dropping funnel at a rate of one to two drops per second. After the reaction was completed, the reaction system was placed in the ice bath for 30 min and then was placed at 25 ℃, during which the

reaction process was tracked through a spot plate. After the reaction was complete, the solvent methanol and excess ethylenediamine were removed by vacuum distillation at 65 °C, the rotated product was dissolved in methanol, and the vacuum distillation was repeated more than three times to obtain a pale yellow viscous liquid (named MA1.0) with a yield of 93.6%.

(3) Under the protection of an ice bath and nitrogen, methyl acrylate solution was dropped into MA1.0 solution by a constant-pressure-dropping funnel with methanol as the solvent. The solution was added at a rate of one to two drops per second and then continued to react in the ice bath for 30 min. After the reaction was completed, the solvent methanol and excess methyl acrylate were removed by vacuum distillation at 45 °C. The product was purified by column chromatography, with silica gel as the adsorbent and ethyl acetate:petroleum ether = 1:10 (volume ratio) as the eluent, and the reaction process was detected by a spot plate at all times to determine the product. Finally, the eluent was removed by vacuum distillation to obtain a yellow viscous liquid (named MA1.5) with a yield of 81.2%.

(4) Under the protection of an ice bath and nitrogen, using dry methanol as solvent, ethylenediamine solution was dripped into MA1.5 solution with a constant-pressure-dropping funnel at a rate of one to two drops per second. After the reaction was completed, placement was continued in the ice bath for 30 min, and then the reaction system was placed at 25 °C, during which the reaction process was detected through a spot plate. After the reaction was completed, the solvent methanol and excess ethylenediamine were removed by vacuum distillation at 65 °C, the rotated product was dissolved in methanol, and the vacuum distillation was repeated more than three times to obtain a dark yellow viscous liquid (named MA2.0) with a yield of 91.7%.

The optimal synthesis conditions for each step of branched monomer are shown in Table S1.

Table S1 Optimal synthesis conditions of branched monomers.

**Reaction product**	**Molar ratio of reaction raw materials**	**Reaction time (h)**	**Reaction temperature (ºC)**
MA0.5	Ethylenediamine:methyl acrylate = 1:8	18.2	
MA1.0	MA0.5:ethylenediamine = 1:24	12.3	25
MA1.5	MA1.0:methyl acrylate = 1:16	32.5	
MA2.0	MA1.5:ethylenediamine = 1:48	21.6	

2. Characterization of branched monomers

2.1 FTIR of branched monomers

The FTIR spectra of the branched monomers are shown in Figure S1. The spectrum of MA0.5 shows the absorption peaks at around 2,957 and 2,833 cm^{-1}, suggesting the stretching vibration of $-CH_3$ and $-CH_2$. The absorption peak near 1,741 cm^{-1} is the characteristic absorption peak of –C = O–, and the absorption peak near 1,446 cm^{-1} is assigned to the characteristic absorption peak of $-CH_3$ for in-plane bending vibration. However, in the spectrum of MA1.0, there are also characteristic absorption peaks of $-NH_2$ (3,280 cm^{-1}) and –CONH– (1,652 cm^{-1}), indicating that MA0.5 has undergone Friedel-Crafts acylation reaction with ethylenediamine. Because the structures of MA0.5 and MA1.5 are similar, the corresponding infrared spectra are also similar. In addition to the same characteristic

absorption peak as that of MA0.5, there are also characteristic absorption peaks of –NH– at 3,301 cm^{-1} and –CONH– at 1,650 cm^{-1} in the spectrum of MA1.5, indicating that MA1.0 has undergone the Michael addition reaction with methyl acrylate. Compared with MA1.5, the absorption peak near 3,278 cm^{-1} was observed in the spectrum of MA2.0, which is the characteristic absorption peak of $–NH_2$. There is no characteristic absorption peak near 1,731 cm^{-1} in the spectrum of MA1.5, indicating that MA1.5 and ethylenediamine have undergone Friedel-Crafts acylation reaction and the target product MA2.0 has been successfully synthesized.

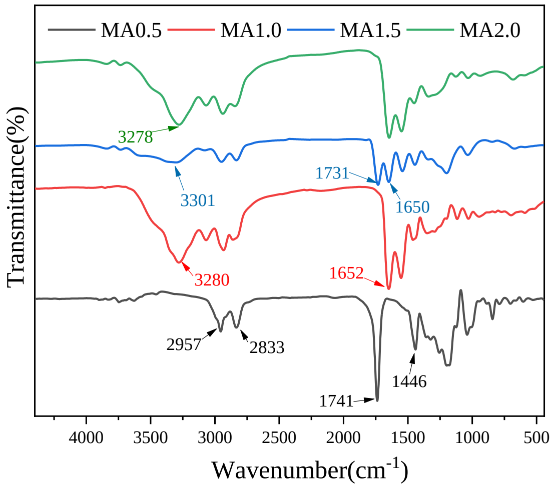

Figure S1. Infrared spectra of MA0.5, MA1.0, MA1.5 and MA2.0

2.2 *^{1}HNMR of branched monomers*

Figure S2 displays the ^{1}HNMR of branched monomers. As can be seen from the ^{1}HNMR spectrum of MA0.5 (Figure S2a), the signal observed at 2.4 to 2.5 ppm was assigned to the protons of $–CH_2–COO–$. The chemical shift values at 2.7 and 3.67 ppm were caused by the protons of $–CH_2$ and $–CH_3$, respectively. Different from the ^{1}HNMR hydrogen spectrogram of MA0.5, the chemical shift of $–CH_3$ disappeared. The peak values at 1.83 and 7.6 ppm were related to the protons of $–NH_2$ and –NH–CO–, respectively. In Figure S2c, the chemical shift values at 2.38 and 2.42 ppm were attributed to the protons of $–CH_2COO–$. The signals of the protons of $–CH_2$ were 2.5 and 2.7 ppm. The chemical shift of 7.2 ppm was related to the protons of –NHCO–. Comparing Figure S2c and Figure S2d, it can be seen that the chemical shift of $–CH_3$ disappeared and the chemical shift of the protons NH_2 appeared in the spectrum of MA2.0, and the amount of hydrogen was corresponded with the result of integration. Therefore, the results of ^{1}HNMR proved the successful modification and synthesis of MA0.5, MA1.0, MA1.5, and MA2.0 monomers, showing that the experimental molecular structure design was reasonable and consistent with the target.

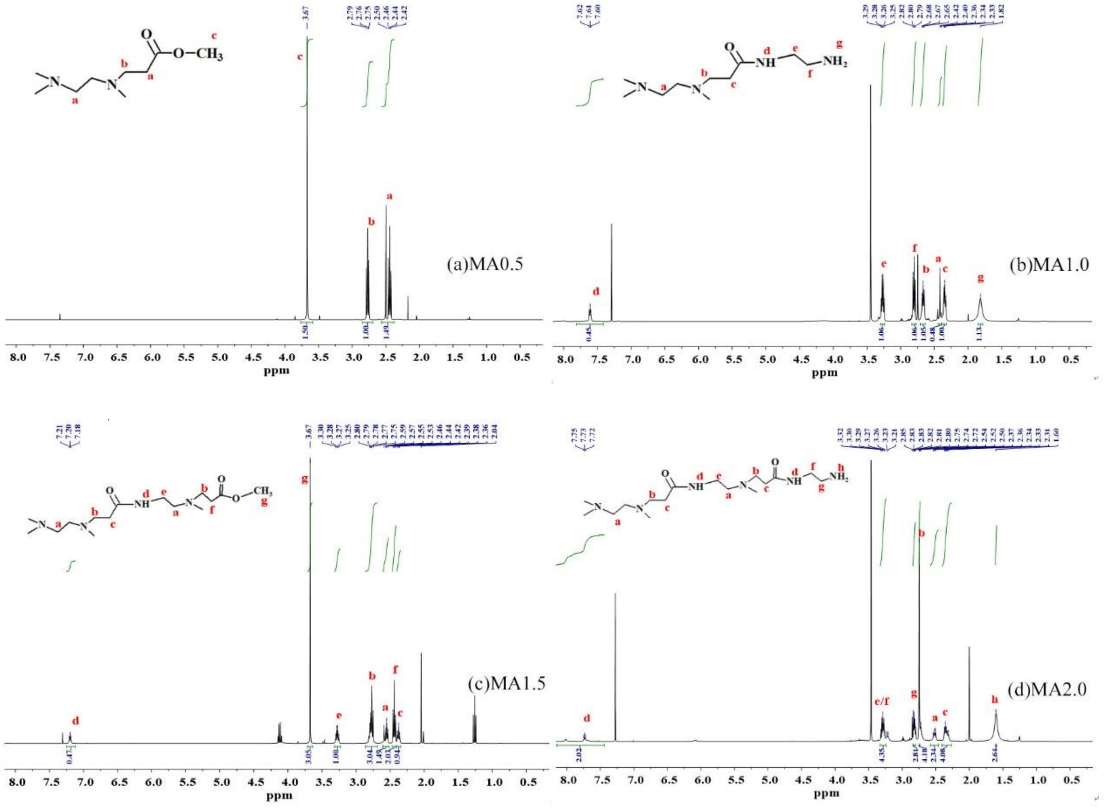

Figure S2. ^{1}HNMR of (a) MA0.5, (b) MA1.0, (c) MA1.5 and (d) MA2.0

3. The synthetic route for each step

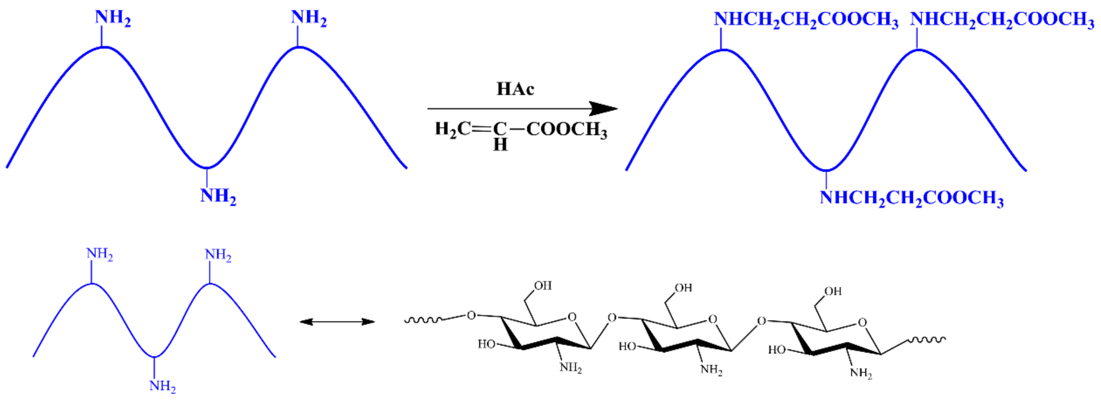

Figure S3. Reaction scheme of NCS

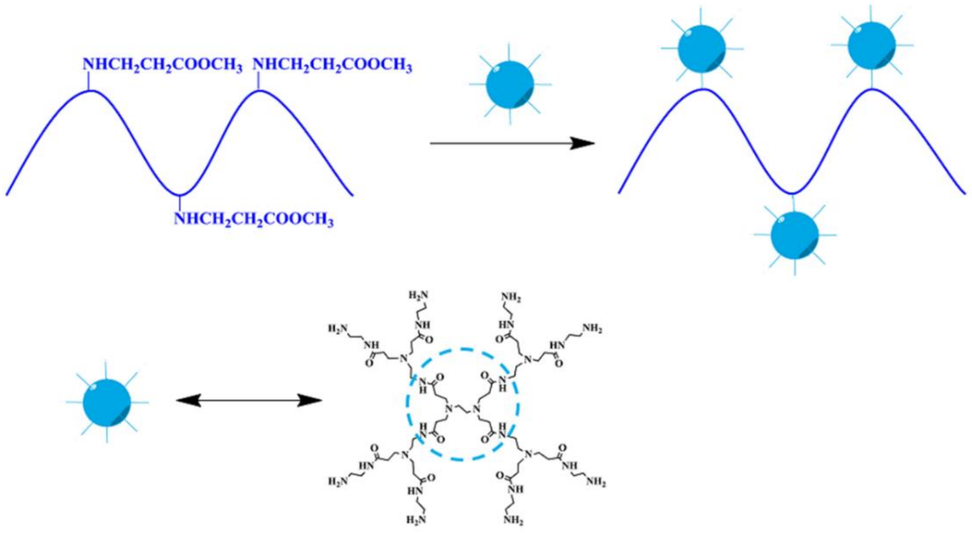

Figure S4. Reaction scheme of MACS

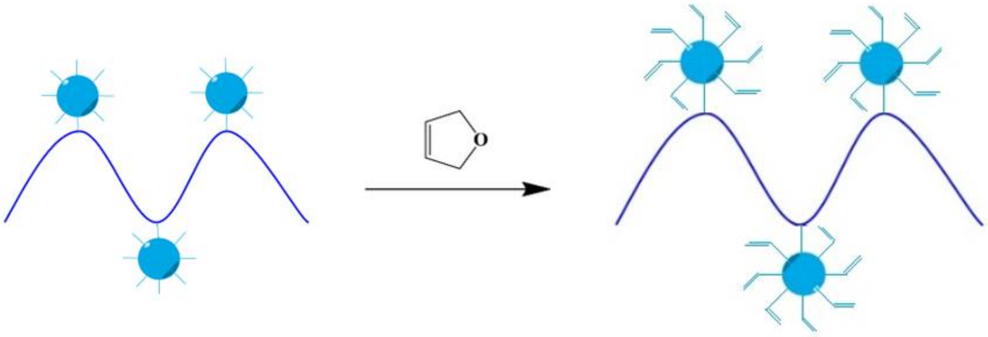

Figure S5. Reaction scheme of DACS

4. Optimization of HPDACS synthesis conditions

The idea to optimize the synthesis conditions of HPDACS is put forward. Firstly, through literature research, the factors and levels affecting the polymerization reaction were roughly determined. Then, on this basis, the corresponding orthogonal test was designed to screen out the influence size and obtain a better level of various factors impacting polymerization reaction. Then, through single-factor experiments, according to the better level determined by the previous orthogonal experiments, each factor was gradually optimized. Finally, through the orthogonal test again, the range was further narrowed and the best synthesis conditions of the polymer were determined.

4.1 Orthogonal test optimization of the better level of each factor

The main factors affecting the polymerization reaction are the total mass concentration of monomers, the mass ratio of AM to AA, the addition of initiator, the addition of functional monomers, and the reaction temperature. Total monomer concentration is 20.0 ~ 32.0 wt.%, mass ratio of AM to AA is 8:2 ~ 5:5, addition of initiator is 0.1 ~ 0.7 wt.%, addition of functional monomer is 0.1 ~ 1.0 wt.%, and reaction temperature is 36.0 ~ 45.0 ℃. Based on this, an orthogonal test of L16 (4^5) was designed to determine the extent of influence and better level of each factor.

The viscosity of the polymer solution was measured by a DV-III rheometer at 60 °C and 7.34 s^{-1} shearing rate. Also, the concentration of the polymer solution was diluted to 100 mg/L, and the BOD_5 of the polymer was measured at 60 °C with a microbial degradation respirator. The test results are shown in Table S2.

Table S2 Orthogonal test table of polymers

No.	Total mass concentration of monomers (wt. %)	AM: AA	Initiator (wt. %)	Functional monomer (wt. %)	Temperature (°C)	Viscosity (mPa·s)	BOD_5 (mg/L)	Score
1	20	8:2	0.1	0.1	36	109.8	676.32	336.41
2	20	7:3	0.3	0.4	39	220.1	2,731.65	1,224.72
3	20	6:4	0.5	0.7	42	288.8	3,016.96	1,390.06
4	20	5:5	0.7	1.0	45	212.2	3,357.29	1,470.24
5	24	8:2	0.3	0.7	45	-	-	-
6	24	7:3	0.1	1.0	42	-	-	-
7	24	6:4	0.7	0.1	39	292.0	826.87	505.95
8	24	5:5	0.5	0.4	36	205.8	2,218.63	1,010.93
9	28	8:2	0.5	1.0	39	-	-	-
10	28	7:3	0.7	0.7	36	159.5	2,879.38	1,247.25
11	28	6:4	0.1	0.4	45	-	-	-
12	28	5:5	0.3	0.1	42	-	-	-
13	32	8:2	0.7	0.4	42	129.2	2,269.77	985.43
14	32	7:3	0.5	0.1	45	-	-	-
15	32	6:4	0.3	1.0	36	-	-	-
16	32	5:5	0.1	0.7	39	-	-	-
k_1	1,105.36	330.46	84.10	210.59	432.65			
k_2	379.22	620.29	306.18	805.27	648.65			
k_3	311.81	474.00	600.25	659.33	539.87			
k_4	246.36	617.99	1,052.22	367.56	367.56			
Range	859.00	289.83	968.11	594.68	281.09			

In this paper, the viscosity of polymer solution and BOD_5 were taken as evaluation indexes, and multiindex orthogonal tests often use a comprehensive scoring method for quantitative treatment. According to the range data converted from the orthogonal test, the optimal level of HPDACS was determined as follows: initiator addition > total monomer concentration > functional monomer addition > AM:AA > reaction temperature. At the same time, the optimal synthesis conditions of each factor were as follows: initiator of 0.7 wt.%, total monomer concentration of 20 wt.%, functional monomer of 0.4 wt.%, AM:AA of 7:3, temperature of 39 °C.

4.2 Single-factor experimental optimization

4.2.1 Optimization of initiator addition

Firstly, the addition of the initiator was optimized, and the range of the amount of addition was changed to 0.4, 0.5, 0.6, 0.7, 0.8, 0.9, and 1.0 wt.%. The synthesis conditions of the other factors were total monomer concentration of 20 wt.%, functional monomer of 0.4 wt.%, AM:AA of 7:3, and temperature of 39 °C. As shown in Figure S6, with the continuous increase of initiator addition, the viscosity and BOD_5 of the polymer solution showed alternating increases and decreases. The comprehensive scoring method was used for quantitative treatment, and the

initiator addition in the polymerization reaction was 0.5 wt.%.

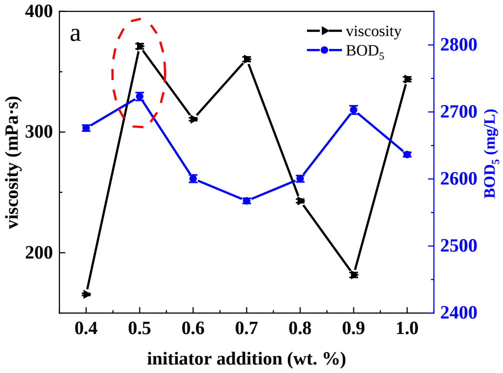

Figure S6. Effect of initiator addition on polymer viscosity and BOD_5

4.2.2 Optimization of total monomer concentration

After 0.5-wt.% initiator was added, the total monomer concentration was optimized to 17, 18, 19, 20, 21, 22, and 23 wt.%. The other synthesis conditions were as follows: functional monomer of 0.4 wt.%, AM:AA of 7:3, and temperature of 39 °C. With the increase of the total monomer concentration, the viscosity of the polymer increased first and then decreased, and the BOD_5 of the polymer showed a trend of increasing first, then decreasing, and then increasing. When the total monomer concentration was 18 wt.%, the viscosity and BOD_5 value of the polymer solution were the largest, so the total monomer concentration of polymerization reaction was 18 wt.%. These results are shown in Figure S7.

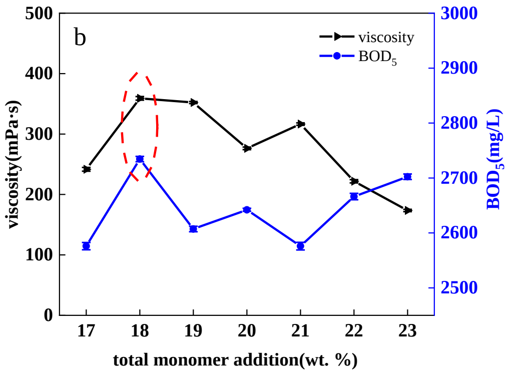

Figure S7. Effect of total monomer concentration on polymer viscosity and BOD_5

4.2.3 Optimization of functional monomer addition

After the total monomer concentration was determined to be 18 wt.%, the addition of functional monomers was optimized, and its range was changed to 17, 18, 19, 20, 21, 22, and 23 wt.%. The synthesis conditions of other factors were as follows: initiator addition of 0.5 wt.%, AM:AA of 7:3, and temperature of 39 °C. As can be seen from Figure S8, with the increase of the addition of a modified chitosan functional monomer, the viscosity of the polymer solution shows a downward trend, while the BOD_5 of the polymer shows an upward trend. Quantitative treatment was carried out by a comprehensive scoring method, and the addition amount of modified chitosan functional monomer in the polymerization reaction was 0.3 wt.%.

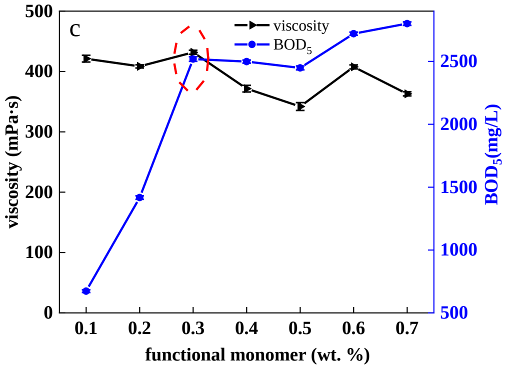

Figure S8. Effect of functional monomer addition on polymer viscosity and BOD_5

4.2.4 Optimization of AM:AA

After the addition of 0.3-wt.% chitosan functional monomers was determined, the mass ratio of AM to AA was optimized to 17, 18, 19, 20, 21, 22, and 23 wt.%. The synthesis conditions of other factors were as follows: addition of initiator of 0.5 wt.%, total concentration of monomers of 18 wt.%, and temperature of 39 °C. After dialysis purification of the synthesized polymer, the concentration required for the experiment was prepared, the viscosity of the polymer solution was measured by a DV-III rheometer, and the BOD_5 of the polymer was measured by microbial degradation respirator. These results are shown in Figure S9.

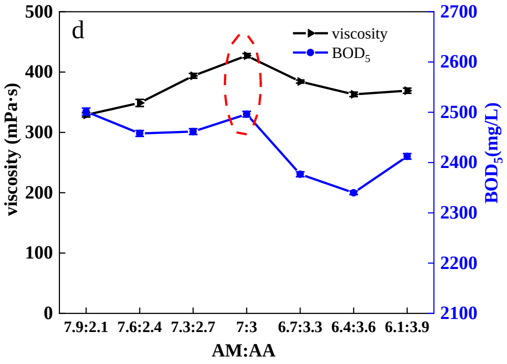

Figure S9. Effect of AM:AA on polymer viscosity and BOD_5

4.2.5 Optimization of reaction temperature

The optimum reaction temperatures were 36, 37, 38, 39, 40, 41, and 42 °C after the mass ratio of AM to AA was 7:3. The reaction conditions were as follows: addition of initiator of 0.5 wt.%, monomer concentration of 18 wt.%, and modified chitosan monomer dosage of 0.3 wt.%. With the increase of reaction temperature, the viscosity of polymer solution increased first and then decreased slightly, and the BOD_5 of the polymer solution increased first, then decreased, and then increased. When the reaction temperature was 39℃, the viscosity and BOD_5 value of the polymer solution were the largest, so the polymerization temperature was 39 °C. These results are shown in Figure S10.

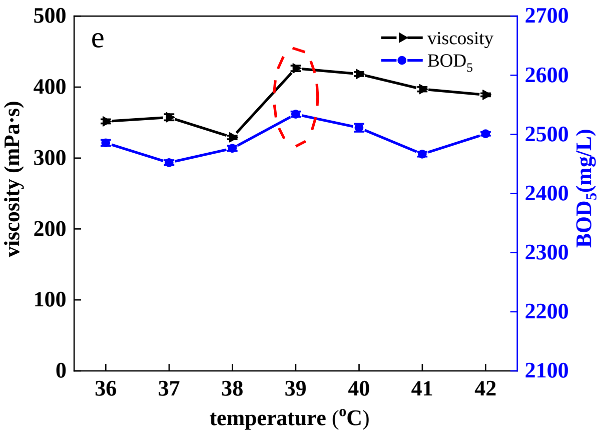

Figure S10. Effect of reaction temperature on polymer viscosity and BOD_5

4.3 Final synthesis condition determined by orthogonal test

Through the single-factor experiments, each factor was gradually optimized. Based on this, through orthogonal tests, the range of various influencing factors was further narrowed, and the best synthesis conditions of the polymer were determined. Among them, the main factors affecting the polymerization reaction were determined to be the total mass concentration of monomer, the mass ratio of AM to AA, the amount of initiator, the amount of functional monomer, and the reaction temperature. The addition of various factors is as follows: total concentration of monomer is 17.6 ~ 18.2 wt.%, mass ratio of AM to AA is 6.8:3.2 ~ 7.1:2.9, addition of initiator is 0.46 ~ 0.52 wt.%, addition of functional monomer is 0.26 ~ 0.32 wt.%, and reaction temperature is 38.6 ~ 39.2 °C. The orthogonal test of L16 (45) was designed to determine the best synthesis conditions of HPDACS.

After 16 groups of polymers were synthesized according to the orthogonal table scheme, HPDACS was prepared into 1,500 mg/L polymer solution with ultrapure water. The orthogonal test results are shown in Table S3.

Table S3 Orthogonal test table of 4^5 polymers

	**Total mass concentration of monomers (wt. %)**	**AM:AA**	**Initiator (wt. %)**	**Functional monomer (wt. %)**	**Temperature (°C)**	**Viscosity (mPa·s)**	**BOD_5 (mg/L)**	**Score**
1	17.6	6.8:3.2	0.46	0.26	38.6	516.4	2,415.7	1,276.1
2	17.6	6.9:3.1	0.48	0.28	38.8	495.7	2,379.2	1,249.1
3	17.6	7:3	0.50	0.30	39	478.1	2,452.9	1,268.0
4	17.6	7.1:2.9	0.52	0.32	39.2	414.3	2,417.3	1,215.5
5	17.8	6.8:3.2	0.48	0.30	39.2	454.2	2,461.4	1,257.1
6	17.8	6.9:3.1	0.46	0.32	39	527.6	2,377.5	1,267.6
7	17.8	7:3	0.52	0.26	38.8	505.2	2,426.8	1,273.8
8	17.8	7.1:2.9	0.50	0.28	38.6	382.4	2,318.6	1,156.9
9	18.0	6.8:3.2	0.50	0.32	38.8	462.2	2,355.8	1,219.6
10	18.0	6.9:3.1	0.52	0.30	38.6	479.7	2,379.8	1,239.7
11	18.0	7:3	0.46	0.28	39.2	403.1	2,402.7	1,202.9
12	18.0	7.1:2.9	0.48	0.26	39	468.5	2,368.9	1,228.7
13	18.2	6.8:3.2	0.52	0.28	39	472.8	2,369.7	1,231.6

14	18.2	6.9:3.1	0.50	0.26	39.2	441.4	2,401.1	1,225.3
15	18.2	7:3	0.48	0.32	38.6	423.9	2,381.6	1,207.0
16	18.2	7.1:2.9	0.46	0.30	38.8	428.7	2,393.7	1,214.7
k1	1,252.18	1,245.43	1,240.33	1,250.95	1,219.93			
k2	1,238.85	1,246.10	1,235.48	1,240.13	1,239.30			
k3	1,222.73	1,237.93	1,217.45	1,244.88	1,248.98			
k4	1,219.65	1,203.95	1,240.15	1,227.43	1,225.20			
Range	32.53	42.15	22.88	40.85	29.05			

Based on this, through the orthogonal test again, the range of various influencing factors was further narrowed, and the optimal level of HPDACS was finally determined as follows: AM:AA > functional monomer addition > total monomer concentration > reaction temperature > initiator addition. At the same time, the optimal synthesis conditions of each factor are as follows: AM:AA of 6.9:3.1, amount of functional monomer of 0.26 wt.%, total monomer concentration of 17.6 wt.%, reaction temperature of 39 °C, and initiator addition of 0.46 wt.%.
